# Incidence and prevalence of multiple sclerosis in Europe: a systematic review

**DOI:** 10.1186/1471-2377-13-128

**Published:** 2013-09-26

**Authors:** Elaine Kingwell, James J Marriott, Nathalie Jetté, Tamara Pringsheim, Naila Makhani, Sarah A Morrow, John D Fisk, Charity Evans, Sarah Gabrielle Béland, Sophie Kulaga, Jonathan Dykeman, Christina Wolfson, Marcus W Koch, Ruth Ann Marrie

**Affiliations:** 1 Division of Neurology, Faculty of Medicine, University of British Columbia, Vancouver, Canada; 2 Department of Internal Medicine, University of Manitoba, Winnipeg, Canada; 3 Department of Community Health Sciences, University of Calgary, Calgary, Canada; 4 Department of Clinical Neurosciences and Hotchkiss Brain Institute, University of Calgary, Calgary, Canada; 5 Institute for Public Health, University of Calgary, Calgary, Canada; 6 Department of Pediatrics, University of Calgary, Calgary, Canada; 7 Division of Neurology and Department of Pediatrics, University of Toronto, Toronto, Canada; 8 Department of Clinical Neurological Sciences, University of Western Ontario, London, Canada; 9 Departments of Psychiatry and Medicine, Dalhousie University, Halifax, Canada; 10 College of Pharmacy and Nutrition, University of Saskatchewan, Saskatoon, Canada; 11 Research Institute of the McGill University Health Centre, McGill University, Montreal, Canada; 12 Department of Epidemiology & Biostatistics and Occupational Health, McGill University, Montreal, Canada

**Keywords:** Multiple sclerosis, Epidemiology, Europe, Incidence, Prevalence

## Abstract

**Background:**

Multiple sclerosis (MS) is the most common cause of neurological disability in young adults worldwide and approximately half of those affected are in Europe. The assessment of differential incidence and prevalence across populations can reveal spatial, temporal and demographic patterns which are important for identifying genetic and environmental factors contributing to MS. However, study methodologies vary and the quality of the methods can influence the estimates. This study aimed to systematically review European studies of incidence and prevalence of MS and to provide a quantitative assessment of their methodological quality.

**Methods:**

A comprehensive literature search was performed to obtain all original population-based studies of MS incidence and prevalence in European populations conducted and published between January 1985 and January 2011. Only peer-reviewed full-text articles published in English or French were included. All abstracts were screened for eligibility and two trained reviewers abstracted the data and graded the quality of each study using a tool specifically designed for this study.

**Results:**

There were 123 studies that met the inclusion criteria. The study estimates were highly heterogeneous, even within regions or countries. Quality was generally higher in the more recent studies, which also tended to use current diagnostic criteria. Prevalence and incidence estimates tended to be higher in the more recent studies and were higher in the Nordic countries and in northern regions of the British Isles. With rare exceptions, prevalence and incidence estimates were higher in women with ratios as high as 3:1. Few studies examined ethnicity. Epidemiological data at the national level was uncommon and there were marked geographical disparities in available data, with large areas of Europe unrepresented and other regions well-represented in the literature. Only 37% of the studies provided standardized estimates.

**Conclusions:**

Despite the breadth of the literature on the epidemiology of MS in Europe, inter-study comparisons are hampered by the lack of standardization. Further research should focus on regions not yet studied and the evaluation of ethnic differences in MS prevalence and incidence. National-level studies using current diagnostic criteria, validated case definitions and similar age- and sex-standardization would allow better geographical comparisons.

## Background

Multiple sclerosis (MS) is a chronic inflammatory disease of the central nervous system that typically presents in the third or fourth decade of life. It is estimated that more than 2 million people have MS worldwide and the disease is among the most common causes of neurological disability in young adults [1]. The distribution and frequency of MS are assessed by estimates of prevalence and incidence. These measures provide essential information for health service planning, and can be used to monitor or reveal spatial, temporal and demographic differences in the distribution of disease. Comparisons of incidence and prevalence in different populations support assessments of the relative contribution of genetic and environmental factors in MS aetiology [2].

MS is recognized worldwide, however reported incidence rates (the proportion of new cases during a defined time period) and prevalence (the proportion of the population that has the disease at or during a specified time) vary considerably between regions and populations [1]. The observed patterns appear consistent with differential genetic predispositions and also implicate environmental risk factors that modulate the risk of MS at the population level [3]. Results of meta-analyses suggest that the incidence of MS has increased over time and provide some evidence that this has primarily resulted from an increase in the incidence of MS among women [4-8]. Europe is considered a high prevalence region for MS (defined by Kurtzke as a prevalence ≥ 30/100,000 [9]), containing more than half of the global population of people diagnosed with MS [1]. Nevertheless, a great deal of uncertainty remains about how the risk of MS varies among European populations. The aim of this study was to systematically review the prevalence and incidence of MS across Europe. The quality of the published studies along with the temporal and geographical trends were examined and priority areas for further epidemiological research identified.

## Methods

### Study selection

This review was part of a larger study on the worldwide incidence and prevalence of MS, which included all original population-based studies published in English or French between January 1st 1985 and January 31st 2011. The start date of 1985 was chosen in part because the introduction of magnetic resonance imaging (MRI) at that time substantially influenced the diagnosis of MS and thus the reliability of case definitions for prevalence and incidence studies. A comprehensive literature search was performed as previously described [10]. The search terms 'multiple sclerosis’, 'incidence’, 'prevalence’ and 'epidemiology’ were entered in MEDLINE and EMBASE databases (see Additional file 1 for detailed search strat-egies), and review articles and bibliographies of original studies so identified were hand searched for potentially relevant studies. Studies in which all data collection was carried out earlier than January 1st 1985 and those that were reported solely as conference presentations or abstracts were excluded.

Two reviewers (RAM, SK or CW) independently screened the abstracts to assess whether each study met all eligibility criteria. If eligibility could not be ascertained by review of the abstract, the full text of the article was reviewed. All articles that met eligibility criteria by consensus of both reviewers were retained.

### Data extraction and quality assessment

For each article, one trained reviewer abstracted data onto a standardized form, including: study location, prevalence day or period, sources for case ascertainment, diagnostic criteria and average age of the study population. Crude and standardized (when available) prevalence and incidence estimates were documented overall and by sex, region, time period and subgroup as applicable. Extracted data were verified by a second reviewer.

Two reviewers independently assessed the quality of each study using a tool designed for this review (Additional file 2) and based on a scoring system suggested by Boyle [1]. The questions aimed to evaluate: the validity of the chosen diagnostic criteria, the representativeness of the study population, the inclusion of confidence intervals, how well the study population was defined, and the reliability and completeness of the data. Each study was scored out of 7 or 8 points based on one potential affirmative score per question. One question applied only to studies that used health administrative data sources; these studies were scored out of 8 while studies using multiple sources of ascertainment were scored out of 7. Conflicts were resolved by consensus. Data abstraction and quality reviews were conducted using the web-based DistillerSR program (Evidence Partners, Ottawa, Canada).

All European studies were then selected to facilitate detailed examination; Russia was included but Turkey, Kazakhstan, Azerbaijan and Georgia were not (these are included in a separate review of studies from Asia; in preparation). The studies were grouped into eight regions to allow more descriptive analysis: the Italian Peninsula and Malta; the British Isles; the Nordic region; the Iberian Peninsula; Belgium and France; the Central European countries; South East Europe; and the Baltic states, including Russia. All data extracted from the European studies were manually verified by one reviewer (EK). Where possible, female to male prevalence and incidence ratios were calculated from reported data whenever sex ratios were not explicitly reported in the manuscripts.

Heterogeneity estimates were generated for the prevalence studies for each European region and for all European studies combined. Studies that did not report either the crude estimate with the confidence interval or the number of cases and the population denominator were excluded from these calculations. We examined the resulting I^2^ statistic, which describes the proportion of variation in point estimates due to heterogeneity between studies rather than to sampling error; a χ^2^ test of homogeneity was conducted to determine the strength of evidence that heterogeneity was genuine.

## Results

**Figure 1:**
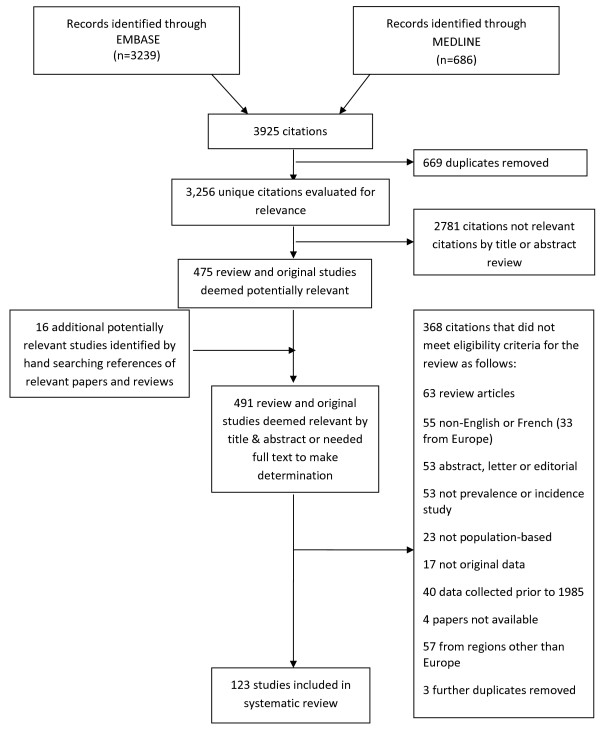
Flow diagram of selection of MS incidence and prevalence studies January 1 1985 – January 31, 2011.

**Figure 2:**
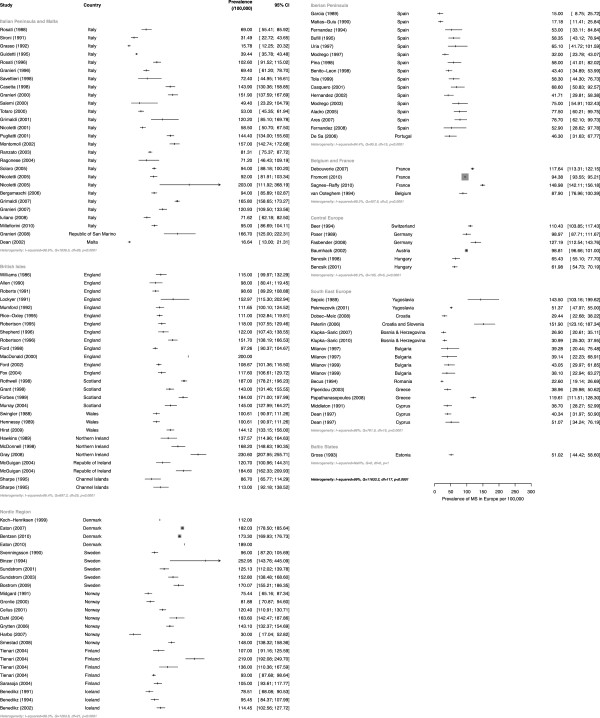
Heterogeneity of prevalence estimates from included studies, stratified by region.

The initial global literature search yielded 3,256 citations through EMBASE and MEDLINE, and a further 16 refer-ences identified by hand searches (see Figure 1). Thirty-three European studies were excluded because of language (eight from Spain, seven from Russia, five from Poland, three Norwegian, three Ukrainian, two German, one Danish, one Czech, one Slovakian, one Serbian and one from the former Yugoslavia). Of the 183 worldwide studies that met the selection criteria, 123 unique studies were conducted in Europe; all data extracted from the European incidence and prevalence studies, with the assessed quality scores, are presented in Additional file 3: Table S1 and Additional file 4: Table S2 respectively (listed chronologically by year of publication, within country). Even when stratified by region, heterogeneity among studies was found to be high (I^2^ ≥ 84.4%, p<0.0001) (see Figure 2). Given the disparity of the studies (I^2^ = 99%, Q =11,633.2, d.f. = 117, p<0.0001), a meta-analysis was not performed.

Prevalence estimates were more frequently reported than incidence estimates; 113 of the 123 studies reported prevalence estimates while 74 reported incidence estimates. Across Europe and over time, point prevalence estimates varied considerably. Estimates as low as ≤ 20/100,000 were reported in some studies conducted in the 1980s [11-14], and also from more recent studies in Malta [15] and in ethnic minority populations of Norway and Bulgaria [16,17]. High estimates of ≥ 200/100,000 were reported in parts of Scotland and Northern Ireland and also within specific populations in Scandinavia [18,19] and Sicily [20]. Likewise, estimated annual incidence rates ranged widely from < 1/100,000 [11,15] to > 10/100,000 [20-22].

In the majority of studies the prevalence of MS was higher in women with sex ratios ranging from 1.1 to 3. The average female to male prevalence ratio overall was approximately 2, ranging from 1.6 for South East Europe to 2.7 for studies from Central Europe; average prevalence ratios across Europe ranged between 1.6 to 2.8 in the 1980s, 1.8 to 2.5 in the 1990s and 1.8 to 2.4 in the 2000s. Exceptions to the excess prevalence of women were noted in a small district of Northern Sweden (female to male ratio: 0.76) where several of the identified cases were related [18], and among Turkish-speaking communities in Cyprus, (female to male ratio: 0.5) [23]. Relatively low female to male prevalence ratios (between 1 and 1.1) were seen in Greek-speaking communities in Cyprus [24], in Catania, Sicily (1989) [13] and in African and Asian ethnic populations in Norway [17]. However, when available, sex incidence ratios revealed generally higher rates for women that were in keeping with the overall higher female sex ratio for prevalence. As incidence ratios are not subject to survivor bias and are more etiologically relevant, their discussion is emphasized in favour of prevalence ratios in the description of the regional findings below.

In most of the reviewed studies cases were ascertained from a variety of sources including hospital and clinic records, neurologists and other physicians, patient associations and, in more recent years, from MS registries or administrative databases. Diagnosis was typically established through assessment by a health professional or review of medical records. MS cases were defined most frequently using the Poser criteria [25] (79% of studies), although the inclusion or exclusion of 'probable’ or 'possible’ MS cases was not consistent across studies. The 2001 McDonald [26], Rose [27] and Schumacher [28] criteria were used in most of the remaining studies while the McAlpine [29] or McDonald/Halliday [30] criteria were used rarely.

Study quality scores varied from 1 to the maximum (7 or 8), and were somewhat lower for earlier studies compared to more recent reports. The mean quality score was 4.31 (standard deviation [SD]: 0.97) for studies conducted during or ending in the 1980s in contrast to 4.86 (SD: 1.02) for those conducted in the 1990s and 5.35 (SD: 1.08) after 2000. Lower quality scores were due to unclear reporting of standardized methods (78% of the studies) or because confidence intervals were not included (25% of the studies). Only 37% of the studies provided standardized estimates, although the diversity of standard populations chosen by the different studies hinders direct comparisons’ of estimates. Nevertheless, estimates that are standardized to a large standard population are preferable to crude estimates and these adjusted estimates, when available, are presented in the following descriptive summaries.

### Italian Peninsula and Malta

Italy has been particularly well-studied, although no single study evaluated the complete Italian mainland. Among the 28 reviewed studies from this region, nine were conducted in Sicily [13,20,31-37], seven in Northern Italy [38-44], seven in Sardinia [45-51], two in Central Italy [52,53], and one each in the south of Italy [54], San Marino [55] and Malta [15]. The Poser diagnostic criteria were used to identify cases in 25/28 of the studies. Prevalence estimates ranged from a low of 15.8/100,000 to a high of 197.8/100,000, with the most extreme variation seen between the studies within Sicily [13,20]. Annual incidence estimates also varied widely across the region, ranging from 0.7 per 100,000 in the Maltese-born population of Malta [15] to 9.2/100,000 in central Sicily [31]. A particularly high incidence estimate of 18.2 per 100,000, for the small town of Linguaglossa, Sicily [20], is thought to represent a geographical and temporal cluster of cases.

It has been suggested that due to different genetic and environmental influences, Sardinia has a higher incidence and prevalence of MS compared to the rest of Italy [56]. Supporting this theory, five of the six studies of the Sardinian population have estimated the prevalence of MS at higher than 100/100,000 [45-47,49,51]. The only study with a lower estimate (69/100,000) was carried out in 1985 [50]. However, when considering the incidence of MS, the Sardinian estimates (3.4 to 6.8/100,000) were not unlike those seen across the entire Italian peninsula.

Female to male ratios for MS incidence tended to be lower in Sicily; ranging from 1.19:1 to 1.84:1 [20,31-34,36] but were as high as 3:1 in San Marino [55] and Northwestern Sardinia [51]. The quality scores ranged between 3/7 and 6/7 with six of the studies from the Italian peninsula scoring 6/7 [33,34,39,51,53]. Ethnicity or race was considered in only one study from this region in which prevalence was reported separately for Maltese-born (16.7/100,000) and foreign-born Maltese (166/100,000) residents [15].

### The British Isles

Together with the Italian peninsula, the British Isles was the most studied region with 28 unique prevalence or incidence studies. Of these, 13 were from England [57-69], six from Scotland [21,70-74], three from Wales [75-77], three from Northern Ireland [78-80], one from the Republic of Ireland [81], and one from the Channel Islands [82]. The remaining study estimated incidence of MS across the UK [83]. Allison and Millar criteria [3] were used, either alone or in combination with Poser criteria, in 12/28 studies from this region conducted between 1985 and 1996 [61,63-65,67,68,70,72,77,79],[80,82].

Prevalence estimates in the British Isles ranged from 96/100,000 in Guernsey [82] to more than 200/100,000, with the highest estimates originating from Scotland [21] and Northern Ireland [78]. These two countries also had the highest annual incidence rates (7.2 to 12.2 per 100,000) [21,78].

Sequential studies of either the same or overlapping populations in the South Glamorgan area of South Wales [75-77], North-Eastern Northern Ireland [78-80], and the Leeds health authority area in England [58,59] all demonstrated increasing prevalence and incidence. For example, in North-Eastern Northern Ireland the prevalence of MS increased from 138/100,000 in the mid-1980s [79] to 200.5/100,000 in 2004 [78].

Annual incidence sex ratios ranged from 1.24:1 in North-Eastern Northern Ireland [78] to 2.82:1 in South-East Wales [76]. The quality scores for studies from the British Isles ranged from 2/7 to 8/8 with seven (25%) of the 28 studies [60,67,71,72,78,81,83] scoring 6 or higher. None of the studies from the British Isles reported prevalence or incidence by ethnic or racial subgroups.

### The Nordic region

Twenty-five studies were reviewed from the Nordic region, including nine from Norway [17,84-91], five from Sweden [18,92-95], four from Denmark [96-99], three from Finland [19,22,100], three from Iceland [101-103] and one from the Faroe Islands [104]. Most (19/25) of the studies used the Poser diagnostic criteria alone or in combination with other criteria. Four studies relied solely on McAlpine [90,91,104] or Schumacher [18] criteria. Administrative International Classification of Diseases (ICD) codes were used to identify MS cases in two serial studies of the Danish National Hospital Register [97,98].

The highest prevalence estimates in the Nordic region (over 200/100,000) were reported in Seinajoki-South, Finland [19] and in a small Northern rural district of Sweden (population denominator 4,744) [18]. Familial factors were suspected to play a role in the high number of cases found in both populations according to the study authors. The lowest prevalence estimates (20 - 30/100,000) in the Nordic countries were documented in Sami, Asian and African ethnic minority groups in Norway, well below the prevalence among 'Western’ Norwegians (170/100,000) during the same time period [17,89]. Prevalence estimates were 150/100,000 or greater in the more recently conducted studies from Norway, Denmark and Sweden [17,85,88,95-98].

Some of the highest annual incidence estimates in the Nordic countries (9.2 and 11.6/100,000) were found in specific central and Western regions of Finland [22,100]. A particularly low incidence was reported for the 1996-2000 time period in Iceland (1.28/100,000) [103]. This may be an underestimate however as it was based on the incidence of MS symptom onset rather than year of diagnosis; additional cases with onset during this period may have been diagnosed after the findings were published in 2002. Annual incidence estimates in Iceland from the 1980s from the same study [103], as well as from a separate earlier study [101], both reported rates similar to that in other Nordic countries (4.1 - 5.3/100,000).

For the 11 studies that reported sex specific incidence or incidence sex ratios, female to male annual incidence ratios ranged between 1.2 and 2.2 with no major regional or temporal differences. Quality scores ranged from 4/7 to 8/8, and seven (28%) of the studies, including all Danish studies, scored 6/7 or higher [86,88,96-99,104]. MS prevalence in ethnic minority groups was investigated in the two Norwegian studies described above [17,89].

### Iberian Peninsula

One study from central Portugal [105] and 15 from Spain were included in the review. The Spanish studies included 11 conducted on the mainland [14,106-115], three from the Canary Islands [12,116,117] and one from the island of Menorca [118]. No studies incorporated the entire country of either Spain or Portugal.

The lowest prevalence estimates originated from the two earliest studies, conducted in the 1980s; from the island of Lanzarote in the Canary Islands (15/100,000) [12] and from the city of Valencia, Spain (17.7/100,0000) [14]. Prevalence increased over time with the highest estimates (72 and 77/100,000) observed in the most recent studies [106,112].

Ten studies from Spain studied annual incidence, reporting values ranging from 2.2 to 5.3/100,000 [14,106,107,111-113,115-118] with the highest estimates from studies concluded after 2000 [106,112,116].

Only two studies reported sex-specific incidence figures. The female to male ratio was 1.73:1 in Menorca [118] and 3.1:1 in Las Palmas City, Gran Canaria [116]. Quality scores ranged from 3/7 to 6/7 with three of the 15 studies (19%) scoring 6/7 [105,107,112]. One study from this region estimated the prevalence of MS in the minority Roma population in Malaga, Southern Spain in 2002 as 52.9/100,000 [109], which was comparable to estimates in the non-Roma population in the same area in 1991 (53/100,000) [110].

### Belgium and France

One prevalence study originated from Belgium [119] and four incidence and/or prevalence studies were from France [120-123]. One French study utilized an administrative database to report national estimates [123].

Prevalence estimates ranged from 80-90/100,000, as observed in Flanders, Belgium [119] and in mostly Southern regions of France [123], up to 120-149/100,000 (2004) across regions of North-East France [123] and including one crude estimate from the Haute-Garonne region in South West France [122]. The overall prevalence of MS in France was estimated at 94.7/100,000 [123].

The lowest reported annual incidence in this region was from the city of Dijon in the mid-1990s (4.3/100,000) [121]. Figures a decade later were higher; 7.5/100,000 for the whole country with regional estimates ranging from 6.1 to 10.8 [123].

Annual incidence sex ratios across France in 2004 demonstrated considerable variation; ranging from 1.4/100,000 in Corsica to 4.1/100,000 in central France [123]. The quality scores for these studies varied between 5/7 and 7/8 with two of the studies (40%) scoring 7/8 [122,123]. No studies examined ethnic sub-groups in either Belgium or France.

### Central European countries

Six studies from Central Europe met selection criteria; one from Switzerland [124], two from Germany [125,126], one from Austria [127] and two from Hungary [128,129]. Crude prevalence estimates from this region ranged from 62/100,000 to 128/100,000. The lowest estimates originated from Hungary in the 1990s [128,129] and from Germany in 1986 [125] while the highest estimate, also from Germany, was found in the most recent study (2006) [126]. Annual incidence was estimated in the studies from Hungary and Germany to range between 6/100,000 and 7.7/100,000 without any clear temporal differences [125,126,128,129].

Two studies provided female to male incidence ratios; a ratio of 3:1 was reported in Germany in 2006 [126] while an earlier study from Hungary completed in 1996 reported a ratio of 1.5:1 [129]. Quality scores in the Central European studies ranged from 1/7 to 6/7 with only one study (17%) scoring 6/7 [126]. No studies from this region reported prevalence or incidence by ethnic or racial subgroups.

### South East Europe

Fourteen of the studies included in this review were from South East Europe, covering the former Yugoslavia [130,131], Croatia [132-134], Slovenia [134], Bosnia and Herzegovina [135,136], Bulgaria [16,137], Romania [11], Greece [138,139], and Greek- and Turkish-speaking communities in Cyprus [23,24].

Most of the prevalence estimates in South East Europe fell between a lower value of approximately 20/100,000, as recorded in Romania [11] and in both rural and urban Roma populations in Bulgaria [16], and an upper value of approximately 50/100,000 in other regional studies [23,24,131,133,135-137,139]. However, higher prevalence estimates (144 and 152/100,000), were documented in the Gorski Kotar region of North-West Croatia [130,134] and in the neighboring region (the municipalities of Kocevje and Ribnica) in South East Slovenia [134]. The one-year incidence estimate for 1986 was also moderately high (3.78/100,000) in these two regions, prompting suspicion that a strong familial influence is at play in this isolated population [130]. A study in western Greece also reported high prevalence (120/100,000) and incidence (9.5/100,000) estimates [138], that were notably higher than those of a study conducted seven years earlier in the northeastern region of Evros (prevalence of 38.9/100,000 and annual incidence of 2.36/100,000) [139]; possibly explained by increased awareness, knowledge and availability of MRI machines [138]. Among the remaining four studies that measured annual incidence of MS, estimates ranged from 0.32/100,000 in Romania [11] and 0.8/100,000 in Croatia [132] to 1.1 or 1.6/100,000 in recent studies from Bosnia and Herzegovina [135,136].

One incidence study provided sex-specific data and reported a female to male ratio of 1.69:1 in Western Greece [138]. Quality scores in South East Europe range from 2/8 to 6/7; only the two studies from Greece [138,139] scoring 6/7. Ethnic differences were highlighted by a 1998 report of MS prevalence among the Roma and non-Roma population in two regions of Bulgaria. The prevalence in the Roma (19/100,000) was found to be half that of the non-Roma (45/100,000) population in both regions [16].

### The Baltic states

Only one study from the Baltic States, a prevalence study in Southern Estonia in 1989, was included [140]. Schumacher criteria were used to identify cases, and the estimated prevalence in the entire population was 50/100,000. The quality score for this study was 4/7. The prevalence of MS in native Estonians was 55/100,000 in contrast to 29/100,000 among those of Russian descent, including those born in Estonia and first-generation Russian immigrants.

## Discussion

This systematic review has comprehensively catalogued the incidence and prevalence of MS across Europe between January 1985 and January 2011, and unlike prior systematic reviews [141-143] of this region, has summarized methodologies and evaluated study quality using an objective measure and a predetermined set of criteria. We aimed to describe potential temporal and demographic patterns that could be appreciated at the continental level and to identify gaps in the literature, including regions or populations that are under-represented.

Some European regions have undergone several MS incidence or prevalence studies over this 27 year period; more than 25 studies originated from each of the British Isles, Italy, and the Nordic region. Spain has also been well represented. Variability in representation within individual countries was marked; of the 28 Italian studies, for example, 16 were performed in either Sardinia or Sicily, and only 12 on the mainland where most of the Italian population lives. There is a relative paucity of studies from Central and Eastern Europe and the 11 Sicilian studies equal the total number of studies undertaken in all of France, Belgium, Germany, Switzerland, Austria and Hungary combined.

While much of the literature has focused on specific regions or individual cities within a given country, a few studies reported countrywide data [15,55,83,96-99,101, 103,123,127]. The extensive population of many European countries limits the capacity to ascertain MS cases at the national level. Administrative databases offer the means to estimate the burden of MS at this level, but comparability between studies has been limited due to the various case definitions that have been used. These have included the granting of permanent disability status with MS or the need for disease modifying therapy [123], the presence of an incident International Classification of Disease code (ICD) for MS [97,98], and the more typical neurologist confirmed diagnosis by standard criteria [83,96]. As validated case definitions for MS in administrative data are now available [144,145], there is the potential for greater comparability between estimates derived from these sources in the future.

Ethnic differences were presented in very few reports. Two Norwegian studies assessed MS rates in Asian and African minority groups, or in the indigenous Sami, separately from the remainder of the Norwegian population [17,89], and found up to an eight-fold lower prevalence in those groups. Non-Maltese born residents (mostly originating from Northern Europe) had a 10-times higher prevalence than Maltese-born individuals [15]. Single studies from Spain [109] and Bulgaria [16] revealed that prevalence was lower in the Roma compared to non-Roma populations from the same regions. Lastly, the Estonian report [140] examined Estonian- and Russian-born populations separately and found a lower prevalence in those originating from Russia. Studies such as these provide unique and valuable information, and can potentially be used to differentiate the role of genetic and environmental factors in MS.

Prevalence and incidence estimates tended to be higher in the Northern regions of the United Kingdom and in the Nordic Countries, implicating the role of latitude. This pattern is not uniform however, with higher estimates originating as far south as Sicily and Greece [20,31,138]. Although there were some rare reports of lower prevalence ratios of women to men [18,23], the incidence sex ratios (when available) revealed consistently higher rates of women than men with MS across Europe with no obvious patterns between north and south. The issues of latitude-dependent gradients in MS incidence, prevalence, and sex ratios, have been addressed in detail by recent reviews [4,146].

The assessed quality of these epidemiological studies varied both geographically and temporally. The more recent literature had higher quality scores in general, with the mean scores increasing from 4.31 for studies with data collection before 1990, to 5.35 in those conducted since 2000. The studies from France and Belgium scored high on average; however, these were also among the most recent. Those originating from the British Isles were methodologically weaker overall but included a greater proportion of earlier studies. When comparing estimates between regions it is important to recognize the inter-related issues of the methodological quality of the study, the size of the source population, the time period over which the study was performed and the diagnostic criteria that were used. For example, the Poser criteria were the most widely used (either alone or combined with other criteria in 100 of the 123 studies), although studies varied regarding inclusion of “probable” and “possible” cases. However, many of the earlier studies from the British Isles relied on the Allison-Millar or Rose criteria; older criteria that may be more inclusive and thereby might inflate prevalence or incidence estimates [69,72]. However, any such effect depends on whether cases in Allison-Millar’s “possible” or “early” categories are included [72,77,80]. The definition of incidence also varied, with most studies reporting incidence based on the date of diagnosis, but others using the date of MS symptom onset [15,39,40,42,46,55,70,87],[101,103,117,118]. This latter definition can sometimes result in an apparent decrease in incidence rates during the most recent time period [103] due to the time-lag between onset and diagnosis [147-149].

The more recent studies reported higher MS prevalence or incidence estimates. Prevalence estimates would be expected to increase over time if life expectancy of those with MS increases; incidence is therefore considered a better indicator of changes in disease rates [4]. However, given the differences in study methodology and quality as described above, it is difficult to determine if the observed changes in incidence estimates over time are due to real changes in the risk of MS. Additional factors which can be related to an earlier diagnosis, including access to neurological care and disease modifying therapies as well as the availability of MRI, have also changed over time. Comparisons are further limited by the lack of appropriate standardization; only 42% of the prevalence studies and 22% of the incidence studies included age- and sex-standardized estimates, and among these, a variety of standard populations were used. The effects of several of these limitations have previously been highlighted and recommendations have been made that would allow for reliable comparisons between MS epidemiological studies [142,143,150].

This review has some limitations. Once the data abstraction of the 123 unique studies from Europe was complete, considerable inter-study variability was evident, preventing a meaningful quantitative synthesis of the data even within regions or countries. The included studies are limited to publications in English or French and, although few studies identified in the initial review were excluded dues to language, their exclusion is likely to have biased data collection in favour of Western European countries. Of the 33 articles excluded for language, 13 originated from countries not represented in this review; i.e. Russia, the Ukraine, Poland and the Czech Republic or Slovakia. The grouping of countries into eight European regions was predominantly based on geography for descriptive purposes, and these groupings may not be appropriate for all questions related to the distribution of MS within Europe. Strengths of the study included the comprehensive assessment of study quality, and the independent data abstraction by two reviewers with subsequent verification by the first author and the comprehensive assessment of study quality. This quality scoring system not only offers a grading system for existing literature but a guide to improving the design of future MS incidence and prevalence studies.

## Conclusion

While there was marked variability in the methodological quality of the studies reviewed, we can report that methods seem to have improved over time, as demonstrated by the trend towards higher quality scores in later studies. Most prevalence and incidence estimates are derived from towns or regions within a country, but national studies have become increasingly feasible with the availability of large databases and registries. The use of such resources may improve comparability between estimates, although attention should be paid to the validity and comparability of case definitions. Spatial and temporal comparisons would be facilitated if studies were to adopt a universal standard population, and if age- and sex-specific estimates were uniformly provided. The prevalence and incidence of MS are not well documented in many regions of Europe. As incidence and prevalence of MS vary considerably between different ethnic populations, greater attention should also be paid to the ethnic composition of source populations and cases.

## Abbreviations

ICD: International classification of diseases; MS: Multiple sclerosis; SD: Standard deviation.

## Competing interests

The authors declare that they have no competing interests.

## Authors’ contributions

EK and JJM participated in the data abstraction, reviewed and verified all abstracted data, drafted and revised the manuscript. NJ and TP conceived of the study and participated in its design and coordination. NM, SM, JF, CE and MWK participated in the data abstraction. SGB and JD participated in the study design and coordination. SK and CW participated in the study design and coordination and reviewed each abstract. RAM conceived of the study, participated in its design and coordination and reviewed each abstract. All authors read and approved the final manuscript.

## Pre-publication history

The pre-publication history for this paper can be accessed here:

http://www.biomedcentral.com/1471-2377/13/128/prepub

## Supplementary Material

Additional file 1 **Multiple sclerosis search strategy EMBASE & MEDLINE.** Details of search strategy to retrieve abstracts. Click here for file

Additional file 2 **Quality assessment form.** Questionnaire completed by two independent reviewers for each study. Click here for file

Additional file 3: Table S1 Incidence of multiple sclerosis, Europe, January 1 1985-January 31, 2011. Click here for file

Additional file 4: Table S2 Prevalence of multiple sclerosis, Europe, January 1 1985-January 31, 2011. Click here for file
