# Supplementary material for: Incidence and prevalence of multiple sclerosis in Europe: a systematic review
Source: BMC Neurol. 2013 Sep 26;13:128. doi: 10.1186/1471-2377-13-128 (PMC3856596; doi:10.1186/1471-2377-13-128)
Supplement: Additional file 1 — Multiple sclerosis search strategy EMBASE & MEDLINE. Details of search strategy to retrieve abstracts. [file 1471-2377-13-128-S1.pdf]

Additional 1. Multiple Sclerosis Search Strategy EMBASE & MEDLINE

Database: EMBASE <1980 to 2011 Week 04>

Search Strategy:

1 exp INCIDENCE/ (200607)

2 exp PREVALENCE/ (256495)

3 *EPIDEMIOLOGY/ (22291)

4 1 or 2 or 3 (455398)

5 exp multiple sclerosis/ (53422)

6 4 and 5 (2163)

7 exp multiple sclerosis/ep [Epidemiology] (2248)

8 6 or 7 (3713)

9 limit 8 to yr="1985 -Current" (3283)

10 limit 9 to animals (44)

11 9 not 10 (3239)

Database: Ovid MEDLINE(R) In-Process & Other Non-Indexed Citations and Ovid MEDLINE(R) <1948 to Present>

Search Strategy:

1 exp Incidence/ (137484)

2 exp Prevalence/ (139838)

3 exp Epidemiology/ (17403)

4 1 or 2 or 3 (280758)

5 exp Multiple Sclerosis/ (35659)

6 4 and 5 (941)

7 exp Multiple Sclerosis/ep [Epidemiology] (2687)

8 6 and 7 (709)

9 limit 8 to yr="1985 -Current" (705)

10 limit 9 to animals (19)

11 9 not 10 (686)
