# Supplementary material for: Incidence and prevalence of multiple sclerosis in Europe: a systematic review
Source: BMC Neurol. 2013 Sep 26;13:128. doi: 10.1186/1471-2377-13-128 (PMC3856596; doi:10.1186/1471-2377-13-128)
Supplement: Additional file 2 — Quality assessment form. Questionnaire completed by two independent reviewers for each study. [file 1471-2377-13-128-S2.pdf]

Additional 2. Quality Assessment Form

Studies are assigned 1 point for each "yes" received on Questions 1-8 for a total score out of 8. If a question is not applicable for the study under review, select "yes".

REPRESENTATIVENESS OF SAMPLE

1. Is the target population clearly defined overall?
e.g. The target population must be defined by shared characteristics assessed and measured accurately. Some of these characteristics include age, sex, ethnicity, income, etc. Clear inclusion and exclusion criteria.

☐ Yes ☐ No ☐ Unclear ☐ Not reported

The following are sub-questions - should all be used to help you determine whether to give an overall "Yes" for the target population (unless do not apply)

A. Are the characteristics of the participants clearly described? ☐ Yes ☐ No

B. Are the inclusion/exclusion criteria clearly described? ☐ Yes ☐ No

C. If answer to 1B above is "Yes", are the inclusion/exclusion criteria appropriate for the question? e.g. are all important groups included. ☐ Yes ☐ No ☐ Not applicable

Comment:

2. Was either of the following methods used to identify members of the target population: 1) probability sampling or 2) entire population surveyed? e.g. Members of the target population were identified through a sampling frame or listing of potential respondents. This listing must provide access to all members of the defined target population except for exclusions acknowledged by the study authors. *N.B. For studies where administrative data were used capturing the whole population, choose "Yes" and select "entire population surveyed". For studies where multiple sources of ascertainment are used (e.g. review of hospital or physician files, MS Society membership lists) you need to decide whether the sources were adequate. If so, (i.e. considered all potentially relevant sources) then mark "Yes" and select "Entire population surveyed". If you do not think that they considered all relevant sources then mark "No"*

☐ Yes ☐ No ☐ Unclear ☐ Not reported

Comment:

3. Is the response rate ≥70%? *N.B. For studies using whole population administrative data mark "Yes". For studies where multiple sources of ascertainment are used mark "Not reported" unless capture-recapture is reported which can be used to estimate "response rate".*

☐ Yes ☐ No ☐ Unclear ☐ Not reported

Comment:

4. Are non-responders clearly described? *N.B. For studies using administrative data, mark "Yes". For studies where multiple sources of ascertainment are used, mark "Not applicable".*

☐ Yes ☐ No ☐ Unclear ☐ Not applicable

Comment:

5. Is the sample representative of the target population? (e.g. need to ensure that non-responders have similar characteristics as responders, otherwise may have selection bias). *N.B. This will be "Yes" for studies using administrative data (i.e if "entire population surveyed" was the response in 2 above). For studies where multiple sources of ascertainment are used if you think they were adequate mark "Yes". If you do not think the case finding process was adequate mark "no".*

☐ Yes ☐ No ☐ Unclear ☐ Not reported

Comment:

ASSESSMENT OF NEUROLOGICAL CONDITION

6. Were data collection methods standardized?
e.g. Identical methods of assessment and data collection were used with all respondents so that the information for analysis is comparable. Standardization of methods refers not only to eliciting information from respondents but also to interviewing training, supervision, enlistment of respondents and processing of data.

☐ Yes ☐ No ☐ Unclear ☐ Not reported

N.B. For administrative data this will be "Yes".

Comment:

7. Were validated criteria used to assess for the presence/absence of disease?
e.g. a validated scale, diagnostic tool, survey, etc. *N.B. For administrative data, this will only be "Yes" if there was validation of the methodology.*

☐ Yes ☐ No ☐ Unclear ☐ Not reported

A. Is the case definition clear? ☐ Yes ☐ No ☐ Not reported
B. Could the case definition be reproduced in another study? ☐ Yes ☐ No ☐ Not clear ☐ Not reported

Comment:

STATISTICAL ANALYSIS

8. Are the estimates of prevalence or incidence given with confidence intervals and in detail by subgroup (if applicable)? *N.B. Mark "Yes" if confidence intervals are given.*

☐ Yes ☐ No ☐ Unclear ☐ Not reported

A. Was the date (for point prevalence) or interval (for period prevalence) presented? ☐ Yes ☐ No ☐ Not clear ☐ Not reported
B. Is type of prevalence clearly mentioned? *N.B. This requires specific use of the terminology period prevalence or point prevalence to get a "Yes"* ☐ Yes ☐ No
C. Were age-specific data presented? ☐ Yes ☐ No
D. Were sex-specific data presented? ☐ Yes ☐ No
E. Were the results standardized to national population? ☐ Yes ☐ No
F. Were confidence intervals provided? ☐ Yes ☐ No
G. Are any covariates considered and measured in the study? ☐ Yes ☐ No
H. Is the statistical analysis appropriate? ☐ Yes ☐ No ☐ Not clear ☐ Not reported
I. Were missing data handled appropriately? (i.e. case deletion, variable deletion, imputation methods)? ☐ Yes ☐ No ☐ Not clear ☐ Not reported

Comment:

TOTAL QUALITY SCORE:

☐ 1 ☐ 2 ☐ 3 ☐ 4 ☐ 5 ☐ 6 ☐ 7 ☐ 8
