# Supplementary material for: Incidence and prevalence of multiple sclerosis in Europe: a systematic review
Source: BMC Neurol. 2013 Sep 26;13:128. doi: 10.1186/1471-2377-13-128 (PMC3856596; doi:10.1186/1471-2377-13-128)
Supplement: Additional file 3: Table S1 — Incidence of multiple sclerosis, Europe, January 1 1985-January 31, 2011. [file 1471-2377-13-128-S3.doc]

## Additional file 3: Table S1 Incidence of Multiple Sclerosis, Europe, January 1 1985-January 31, 2011

| **Ref#** | **Quality Score** | **Study Region, Interval period Sources** | **Diagnostic Criteria, How Established** | **Number cases** | **Type of Incidence Reported** | **Population Denominator** | **Crude Incidence [Standardized Incidence] Men** | **Crude Incidence [Standardized Incidence] Women** | **Crude Overall Incidence*** | **Age-Standardized Overall Incidence*** | **Female: male ratio** |
| --- | --- | --- | --- | --- | --- | --- | --- | --- | --- | --- | --- |
| **Italy** | | | | | | | | | | | |
| [50] | 5/7 | Town of Sassari, Sardinia 1965—1985  AD/HC/NP/PA | RO CA | 79 | Mean annual incidence | 111,598 | 2.2 (1.4-3.2)  [—] | 4.5 (3.4-5.8)  [—] | 3.4 (2.7-4.2) | — | 2.05 |
| [43] | 4/7 | Valle d'Aosta  1971—1985  HC/IT/NP/PA | PD/PP CA/CR | 36 | Mean annual incidence | 114,325 | —  [—] | —  [—] | 2.1 | — | — |
| [41] | 5/7 | Provinces of Reggio Emilia and Modena, Emilia-Romagna Region  1985—1990  HC/LTC/NP/PA | McA  CA/CR | 105 | Mean annual incidence | 1,014,407 | —  [—] | —  [—] | 1.73 (1.42-2.10) | — | — |
|  |  | Province of Reggio Emilia  1985—1990  HC/LTC/NP/PA |  | 45 |  | 416,088 | —  [—] | —  [—] | 1.8 (2.42-1.31) | — | — |
|  |  | Province of Modena  1985—1990  HC/LTC/NP/PA |  | 60 |  | 598,319 | —  [—] | —  [—] | 1.67 (1.28-2.16) | — | — |
| [51] | 6/7 | Northwestern Sardinia  1987—1991  HC/NP/R | PD/PP  CA/CSF/IT | 75 | Mean annual incidence | 268,926 | 2.8  [—] | 8.4  [—] | 5.7 (4.5-7.1) | — | 3.00 |
| [40] | 5/7 | Province of Ferrara  Emilia-Romagna Region  1965—1993  AD/HC/IT/NP/PA/R | PD/PP  CA/CR | 252 | Mean annual incidence | 381,118 | 1.5 (1.2-1.9)  [1.6] | 3.0 (2.6-3.5)  [3.2] | 2.3 (2.0-2.6) | 2.4 | 2.00 |
| [37] | 5/7 | City of Monreale, Sicily  1981—1991  HC/MR/R | PD  CA/CR | 11 | Mean annual incidence | 25,065 | —  [—] | —  [—] | 3.3 | — | — |
| [36] | 5/7 | City of Bagheria, Sicily  1985—1994  HC/MC/MR/NP/R | PD  CA/CR | 20 | Mean annual incidence | 45,338 | 3.1  [—] | 5.7  [—] | 4.4 (2.7-6.8) | — | 1.84 |
| [46] | 5/7 | Area of Barbagia, Nuoro, Sardinia  1955—1995  AD/HC/NHS/NP/R | PD/PP  CA/CR | 469 | Mean annual incidence | 273,248 | 2.83 (2.41-3.3)  [2.98] | 5.52 (4.9-6.18)  [5.42] | 4.18 (3.8-4.62) | 4.3 | 1.95 |
|  |  | Area of Barbagia, Nuoro, Sardinia  1990—1995  AD/HC/NHS/NP/R |  | 104 |  | — | —  [—] | —  [—] | 6.4 (5.25-7.8) | — | — |
| [32] | 5/7 | City of Enna, Sicily  1986—1995  HC/MC/NP/PA/R | PD  NR | 16 | Mean annual incidence | 28,273 | 5.1  [—] | 6.1  [—] | 5.7 (2.0-10.5) | — | 1.20 |
| [49] | 5/7 | Province of Sassari, Sardinia  1993—1997  AD/HC/NP/PA/R | PD/PP  CA | 158 | Mean annual incidence | — | 4.2 (3.2-5.6)  [—] | 9.2 (7.6-11.1)  [—] | 6.8 (5.8-7.9) | — | 2.19 |
| [33] | 6/7 | City of Catania, Sicily  1990—1994  HC/NP/PA | PD/PP  CA/CR | 66 | Mean annual incidence | 337,964 | 3.2 (2.1-4.7)  [—] | 4.5 (3.2-6.1)  [—] | 3.9 (3.0-5.0) | — | 1.41 |
|  |  | City of Catania, Sicily  1975—1994  HC/NP/PA |  | 170 |  | 374,049 | 2.1 (1.6-2.6) | 2.5 (2.0-3.0) | 2.3 (2.0-2.6) |  | 1.19 |
| [42] | 5/7 | Province of Padova  Lombardy region  1980—1999  AD/HC/NHS/NP/PA | PD/PP  CR | 580 | Mean annual incidence | 820,000 | 2.68 (2.21-3.15)  [—] | 4.45 (3.72-5.18)  [—] | 3.5 (3.03-3.97) | 3.5 | 1.66 |
|  |  | Province of Padova  Lombardy region  1995—1999  AD/HC/NHS/NP/PA |  | — |  | — | —  [—] | —  [—] | 4.2 (3.74-4.66) | — | — |
| [35] | 5/7 | City of Monreale, Sicily  1992—2000  HC/NP/PA | PD/PP  CR | 10 | Mean annual incidence | 27,874 | —  [—] | —  [—] | 4.0 (1.9-6.1) | — | — |
| [34] | 6/7 | City of Catania, Sicily  1995—1999  AD/HC/LTC/MR/NP | PD/PP  CA/CR | 155 | Mean annual incidence | 331,509 | 3.5 (2.7-4.5)  [—] | 5.7 (4.6-6.9)  [—] | 4.7 (4.0-5.5) | — | 1.63 |
| [20] | 5/7 | Town of Linguaglossa,  Catania, Sicily  1991—2000  AD/HC/LTC/NP/PA/R | PD/PP  CA/CR | 10 | Mean annual incidence | 5,491 | 15.4 (4.2-39.4)  [—] | 21.2 (7.8-46.1)  [—] | 18.2 (5.9-42.5) | — | 1.38 |
| [48] | 5/7 | Province of Sassari, Sardinia  1995—1999  MS/R | PNS  CA/CR | 139 | Mean annual incidence | 453,628 | —  3.8 | —  7.6 | 6.1 (5.1-7.2) | 5.8 | 2.20 |
|  |  | Province of Sassari, Sardinia  1965—1999  MS/R |  | 689 |  | 425,442 | —  2.6 | —  6.1 | 4.6 (4.2-4.9) | 4.4 | 2.60 |
| [31] | 5/7 | City of Caltanissetta, Sicily  1993—2002  HC/NP/PA | PD  NR | 56 | Mean annual incidence | 60,919 | 6.6  [—] | 11.5  [—] | 9.2 (8.4-10.0) | — | 1.74 |
| [39] | 6/7 | Province of Ferrara  Emilia-Romagna Region  1990—2003  AD/HC/MR/NP/PA/R | PD/PP  CA/CR | 200 | Mean annual incidence | 365,983 | 2.63 (1.99-3.39)  [2.56] | 5.91 (4.98-6.96)  [5.91] | 4.35 (3.77-4.99) | 4.28 | 2.25 |
| [54] | 3/7 | Province of Salerno  Campania region  2001—2005  R | PNS  CR | 56 | Mean annual incidence | 258,841 | —  [—] | —  [—] | 4.32 (3.24-5.6) | — | — |
| **Republic of San Marino** | | | | | | | | | | | |
| [55] | 5/7 | Republic of San Marino  1990—2005  AD/HC/MR/NP | PD/PP  CR | 33 | Mean annual incidence | 26,184 | 3.9 (1.7-7.7)  [—] | 11.7 (7.6-17.3)  [—] | 7.9 (5.3-11.1) | 7.7 | 3.00 |
| **Malta** | | | | | | | | | | | |
| [15] | 4/7 | Malta (Maltese-born residents)  1979—1998  CSF/HC/LTC/MR/NP/R | PD/PP  CR | — | Mean annual incidence | — | —  [—] | —  [—] | 0.7 | — | — |
| **United Kingdom** | | | | | | | | | | | |
| [83] | 8/8 | United Kingdom  1993—2000  AD | McD/PD/PP  CA/CR/IT | 642 | Mean annual incidence | 1,468,205 | 3.4 (3.0-3.9)  [3.1 (2.6-3.5)] | 7.4 (6.8-8.1)  [7.2 (6.5-7.8)] | 5.5 (5.1-5.9) | 5.1 | 2.5 |
| **England** | | | | | | | | | | | |
| [63] | 5/7 | Cambridge Health District  1^st^ Jul 1990—1^st^ Sep 1991  HC/LTC/NP/PA | AM/PD/PO/PP  CA/CR | 20 | Mean annual incidence | 288,410 | —  [—] | —  [—] | 5.9 (3.9-10) | — | — |
| [67] | 6/7 | Rochdale Metropolitan Borough  1979—1988  AD/HC/NP/PA | AM/PD/PO/PP  CA/CR | — | Annual | — | —  [—] | —  [—] | 5.4 | — | — |
| [66] | 5/7 | South Cambridgeshire  1990—1994  HC/LTC/NP/R | AM/PD/PO/PP  CR/PI | 96 | Mean annual incidence | 290,700 | —  [—] | —  [—] | 6.6 | — | — |
| [58] | 5/7 | Leeds Health Authority  1^st^ Nov1995—31^st^ Dec 1996  HC/LTC /NP/PA | PD/PO/PP  CR | 40 | Annual incidence | 732,061 | —  [—] | —  [—] | 4.4 | — | — |
| [62] | 4/7 | London  1995—1^st^ July 1996  AD/HC/NP | NR  CR | — | Mean annual incidence | 100,230 | —  [—] | —  [—] | — | 7 (4-11) | — |
| [59] | 5/7 | Leeds Health Authority  1996—1998  MS/NP/PA | PD/PO/PP  CR | 130 | Mean annual incidence | 728,840 | —  [—] | —  [—] | 6.1 (5.1-7.2) | — | 2.3 |
| **Scotland** | | | | | | | | | | | |
| [70] | 4/7 | Shetland Islands  1974—23 Feb 1986  HC/NP/PA/R | AM  CR | — | Annual (1985) | 23,454 | —  [—] | —  [—] | 4 | — | — |
| [21] | 5/7 | Southeast Scotland  1989 —1995  HC/LTC/MR/NP | PD/PO/PP  CA/CR | 310 | Mean annual incidence | 864,300 | —  [—] | —  [—] | 12.0 (10.6-13.3) | — | 2.3 |
|  |  | Lothian Region  1989 —1995  HC/LTC/MR/NP |  | — |  | 758,600 | —  [—] | —  [—] | 12.2 (10.8-13.7) | — | — |
|  |  | Border Region  1989 —1995  HC/LTC/MR/NP |  | — |  | 105,700 | —  [—] | —  [—] | 10.1 (6.6-13.6) | — | — |
| [74] | 5/7 | Glasgow  1989—1998  HC/NP | DN  CR | 96 | Mean annual incidence | 169,000 | 3.5 (2.2-4.7)  [—] | 7.7 (7.1-8.3)  [—] | 5.7 (4.5-6.8) | — | 2.2 |
| [71] | 7/7 | Tayside Region  1970—1997  HC/NHS/NP | PD/PP  NR | 772 | Mean annual incidence | 383,405 | —  [—] | —  [—] | 7.2 (4.5-9.9) | — | — |
| **Wales** | | | | | | | | | | | |
| [75] | 4/7 | South East Wales  1985—1988  AD/HC/NP/PA | PD/ PP  CR | 61 | Mean annual incidence | 376,718 | —  [—] | —  [—] | **5.4** | — | 2.00 |
| [76] | 5/7 | South East Wales  1985—2007  AD/HC/NP | McD/PD/PP  CA/CR | 582 | Mean annual incidence | 424,633 | 1.65  [—] | 4.66  [—] | 6.31 | — | 2.82 |
| **Northern Ireland** | | | | | | | | | | | |
| [78] | 6/7 | Ballymoney, Coleraine, Ballymena and Moyle districts  1996  AD/HC/MS/NP/PA | McD/PD/PP  AD/CA/CR | 14 | 1-year incidence | 151,000 | 8.3  [—] | 10.3  [—] | 9.3 | — | 1.24 |
| **Denmark** | | | | | | | | | | | |
| [99] | 8/8 | Denmark  1980—1989  HC/NP/R | AM/PD/PO/PP  CA/CR/IT/NHS | — | Mean annual incidence | — | —  [—] | —  [—] | 4.99 (4.78-5.23) | — | — |
| **Faroe Islands** | | | | | | | | | | | |
| [104] | 6/7 | Faroe Islands  1986—2007  HC/NP | McD/PD/PP  CA/CR/CSF/IT/MR | 43 | Mean annual incidence | 46,983 | 3.3 (1.9-5.3)  [—] | 4.9 (2.4-6.0)  [—] | 4.5 (3.1-6.0) | — | 1.48 |
| **Sweden** | | | | | | | | | | | |
| [94] | 5/7 | Gothenburg region  1983—1988  CSF/HC/NP/R | PD/PO/PP  AD/CR | — | Mean annual incidence | — | —  [1.6] | —  [2.4] | — | 2.0 (1.6-2.4) | 1.50 |
| [93] | 5/7 | Vasterbotten  1988—1997  AD/CSF/HC/LTC/NP/R | PD/PP  AD/CR | 133 | Mean annual incidence | 259,163 | 3.7 (2.7-4.9)  [—] | 6.7 (6.0-8.3)  [—] | 5.2 (4.4-6.2) | — | 1.81 |
| [95] | 5/7 | County of Varmland  1996—2000  HC/NP | PD/PP  CA/CR | 89 | Mean annual incidence | 278,448 | 3.91 (2.26-5.09)  [—] | 8.83 (6.99-11.43)  [—] | 6.39 (5.06-7.72) | — | 2.26 |
| **Norway** | | | | | | | | | | | |
| [87] | 4/7 | Hordaland County  1983—1987  HC/R | McA  NR | 64 | Mean annual incidence | 405,063 | —  [—] | —  [—] | 3.2 | 3.39 | 1.20 |
| [91] | 4/7 | More & Romsdal County  1985—1991  AD/HC/NHS/NP/PA/R | McA  CR | 66 | Mean annual incidence | 238,278 | 2.81  [—] | 5.04  [—] | 4.01 | — | 1.79 |
| [86] | 6/7 | Troms and Finnmark  1989—1992  AD/HC | PD/PP/R  CA/CR/CSF/IT | 38 | Mean annual incidence | 224,724 | —  [—] | —  [—] | 4.3 (3.0-5.9) | — |  |
| [84] | 5/7 | Oslo  1992—1996  AD/HC/LTC/NP/PA | PD  CA/CR/CSF/IT/MR | 210 | Mean annual incidence | 483,617 | —  [—] | —  [—] | 8.7 (6.3-11.9) | — | — |
| [85] | 5/7 | Nord-Trondelag County  1994—1998  AD/HC/MC/NHS/NP | PD/PP  CA/CR | 34 | Mean annual incidence | 127,108 | 4.4 (2.4-7.4)  [—] | 6.3 (3.9-9.7)  [—] | 5.3 (3.7-7.5) | 5.6 | 1.43 |
| [88] | 6/7 | Hordaland County  1998—2002  HC/R | PD/PP  CR | 65 | Mean annual incidence | 435,167 | 2.7 (1.8-3.9)  [—] | 3.3 (2.3-4.6)  [—] | 3 (2.3-3.8) | 3.0 | 1.22 |
| [17] | 5/7 | Oslo (all residents)  2001—2005  NP/R | PD  NR | 172 | Mean annual incidence | 518,090 | —  [—] | —  [—] | 6.6 (5.7-7.7) | — | — |
|  |  | Oslo (Norwegian/Western descent)  2001—2005  NP/R |  | 161 |  | 431,471 | —  [—] | —  [—] | 7.5 (6.4-8.7) | — | — |
| **Finland** | | | | | | | | | | | |
| [22] | 5/7 | Uusimaa  1979—1993  AD/HC | PD  AD/CR | 736 | Mean annual incidence | 1,277,932 | 3.1  [—] | 7.0  [—] | 5.1 (4.8-5.5) | — | 2.26 |
|  |  | Seinajoki  1979—1993  AD/HC |  | 240 |  | 197,042 | 9.1  [—] | 13.6  [—] | 11.6 (10.1-13.1) | — | 1.49 |
|  |  | Vaasa  1979—1993  AD/HC |  | 90 |  | 179,079 | 3.4  [—] | 7.0  [—] | 5.2 (10.1-13.1) | — | 2.06 |
| [100] | 5/7 | Central Finland  1994—1998  HC/CR | PD  AD/CR | 105 | Mean annual incidence | — | 5.5 (3.6-7.5  [—] | 12.6 (9.8-15.5)  [—] | 9.2 (7.4-10.9) | — | 2.29 |
|  |  | Central Finland  1979—1998  HC/CR |  | 231 |  | — | 3.3 (2.6-41.1)  [—] | 7.0 (5.9-8.1)  [—] | 5.2 (4.5-5.9)  [—] | — | 2.20 |
| **Iceland** | | | | | | | | | | | |
| [101] | 4/7 | Iceland  NP/PA  1984—1989 (MS onset years) | PNS  CA | — | Mean annual incidence | 264,000 | —  [—] | —  [—] | 3.5 | — | — |
| [103] | 4/7 | Iceland  NP/PA  1996—2000 (MS onset years) | PNS  CA | — | Mean annual incidence | 278,717 | —  [—] | —  [—] | 1.28 | — | — |
|  |  | Iceland  NP/PA  1991—1995(MS onset years) |  | — |  | 253,500 | —  [—] | —  [—] | 3.71 | — | — |
|  |  | Iceland  NP/PA  1986—1990 (MS onset years) |  | — |  | — | —  [—] | —  [—] | 5.28 | — | — |
| **Spain** | | | | | | | | | | | |
| [14] | 4/7 | Alcoy health region, Valencia  Aug 1986—Aug 1988  HC/MC/NP/PA | R  CA | 6 | Mean annual incidence | 133,915 | —  [—] | —  [—] | 2.24 | — | — |
| [115] | 5/7 | Gijon City, Asturias  1987—1995  HC/LTC/M/MR/NHS/NP/PA | PD/PP  HC | 10 | Mean annual incidence | 33,775 | —  [—] | —  [—] | 3.7 (1.4-7.0) | — | — |
| [111] | 5/7 | Teruel  1991—1996  HC/NP/PA/R | PD/PP  CA | — | Mean annual incidence | — | —  [—] | —  [—] | 2.2 | — | — |
| [113] | 5/7 | Sanitary District of Calatayud  1980—1989  HC/MC/NP/PA | PD  CA | — | Mean annual incidence | — | —  [—] | —  [—] | 2.6 | — | — |
| [107] | 6/7 | Mostoles  1994—1998  HC/NHS/NP/PA | PD/PP  CA/CR | 38 | Mean annual incidence | 195,979 | —  [—] | —  [—] | 3.8 (2.7-5.3) | — | — |
| [118] | 5/7 | Menorca  1987—1996  HC/NP/PA | PD/PP  CA | 22 | Mean annual incidence | 67,009 | 2.82 (0.8-4.3)  [—] | 4.9 (2.9-8.2)  [—] | 3.4 (2.2-5.3) | — | 1.74 |
| [117] | 3/7 | La Palma, Canary Islands  1995—1998  HC/NP/PA | PD/PP  CA/CR | — | “period of study” | 81,507 | —  [—] | —  [—] | 2.25 | — | — |
| [112] | 6/7 | Bajo Aragon, Teruel  1994—2002  R | PD/PP  CA | 24 | Mean annual incidence | 58,666 | —  [—] | —  [—] | 4.6 (2.8-6.5) | — | — |
| [116] | 5/7 | Las Palmas City, Gran Canaria, Canary Islands  1998—2002  AD/HC/MC/MR/NHS/NP/PA | McD/PD/PP  CA/CF | 17 | Mean annual incidence | 413,115 | 2.0  [—] | 6.2  [—] | 4.1 (2.4-6.6) | — | 3.10 |
| [106] | 5/7 | Galicia, Santiago de Compostela  1998—2003  HC/MC/NP/PA | PD/PP  CA | — | “per year” | — | —  [—] | —  [—] | 5.3 (3.19-7.46) | — | — |
| **France** | | | | | | | | | | | |
| [121] | 5/7 | Dijon  1993—1997  HC/MR/NP/R | PD/PP  CR | 21 |  | 95,616 | 2.8 (1.21-6.29)  [—] | 5.9 (3.7-7.15)  [—] | 4.3 (2.91-7.18) | — | 2.11 |
| [120] | 5/7 | Lorraine  1990—2002  HC/NP/PA/R | PD/PP  MC | 1,658 | Mean annual incidence | 2,310,376 | —  3.2 (2.4-4.0) | —  7.6 (5.9-9.3) | — | 5.5 (4.4-6.6) | 2.38 |
| [123] | 7/8 | Metropolitan France  31^st^ Oct 2003—31^st^ Oct 2004  AD | AD  CA | 4,032 | 1-year | 52,359,912 | 4.2 (4.0-4.3)  [—] | 10.4 (10.2-10.6)  [—] | 7.5 (7.3-7.6) | — | 2.48 |
|  |  | Lorraine  31^st^ Oct 2003—31^st^ Oct 2004  AD |  | 227 |  | 2, 008, 606 | —  6.2 (5.4-7.0) | —  15.0 (13.8-16.2) | — | 10.8 (10.1-11.6) | 2.42 |
|  |  | Champagne Ardenne  31^st^ Oct 2003—31^st^ Oct 2004  AD |  | 100 |  | 1,107,627 | —  4.8 (3.8-5.8) | —  12.6 (11.1-14.1) | — | 8.9 (8.0-9.8) | 2.63 |
|  |  | Picardie  31^st^ Oct 2003—31^st^ Oct 2004  AD |  | 181 |  | 1,614,238 | —  6.0 (5.1-6.9) | —  15.6 (14.3-17.0) | — | 11 (10.2-11.8) | 2.60 |
|  |  | Bourgogne  31^st^ Oct 2003—31^st^ Oct 2004  AD |  | 90 |  | 1,330,427 | —  3.1 (2.4-3.8) | —  10.0 (8.8-11.2) | — | 6.8 (6.0-7.5) | 3.23 |
|  |  | Franche Comte  31^st^ Oct 2003—31^st^ Oct 2004  AD |  | 85 |  | 973,046 | —  3.7 (2.8-4.6) | —  13.2 (11.6-14.8) | — | 8.6 (7.7-9.6) | 3.57 |
|  |  | Nord pas de Calais  31^st^ Oct 2003—31^st^ Oct 2004  AD |  | 289 |  | 3,627,506 | —  4.7 (4.1-5.2) | —  10.5 (9.8-11.2) | — | 7.7 (7.2-8.1) | 2.23 |
|  |  | Alsace  31^st^ Oct 2003—31^st^ Oct 2004  AD |  | 161 |  | 1,597,543 | —  5.9 (3.1-6.8) | —  13.2 (11.9-14.4) | — | 9.7 (8.9-10.5) | 2.24 |
|  |  | Centre  31^st^ Oct 2003—31^st^ Oct 2004  AD |  | 188 |  | 2,077,615 | —  5.2 (4.5-6.0) | —  12.4 (11.3-13.4) | — | 9.0 (8.3-9.6) | 2.38 |
|  |  | Auvergne  31^st^ Oct 2003—31^st^ Oct 2004  AD |  | 89 |  | 1,065,898 | —  4.1 (3.2-5.0) | —  11.7 (10.3-13.2) | — | 8.1 (7.3-9.0) | 2.85 |
|  |  | Basse Normandie  31^st^ Oct 2003—31^st^ Oct 2004  AD |  | 83 |  | 1,179,272 | —  3.7 (2.8-4.5) | —  10.0 (8.8-11.3) | — | 7.0 (6.3-7.8) | 2.70 |
|  |  | Haute Normandie  31^st^ Oct 2003—31^st^ Oct 2004  AD |  | 132 |  | 1,619,807 | —  4.4 (3.7-5.2) | —  11.2 (10.1-12.4) | — | 8.0 (7.3-8.7) | 2.55 |
|  |  | Bretagne  31^st^ Oct 2003—31^st^ Oct 2004  AD |  | 196 |  | 2,381,323 | —  4.3 (3.7-4.9) | —  11.4 (10.5-12.3) | — | 8.1 (7.5-8.6) | 2.65 |
|  |  | Limousin  31^st^ Oct 2003—31^st^ Oct 2004  AD |  | 39 |  | 556,268 | —  2.6 (1.6-3.6) | —  10.6 (8.7-12.4) | — | 6.9 (5.8-8.0) | 4.08 |
|  |  | Aquitaine  31^st^ Oct 2003—31^st^ Oct 2004  AD |  | 153 |  | 2,416,622 | —  3.0 (2.5-3.6) | —  8.9 (8.1-9.7) | — | 6.2 (5.7-6.7) | 2.97 |
|  |  | Midi Pyrenees  31^st^ Oct 2003—31^st^ Oct 2004  AD |  | 158 |  | 2,158,845 | —  4.5 (3.9-5.2) | —  9.2 (8.3-10.1) | — | 7.0 (6.4-7.5) | 2.04 |
|  |  | Rhone Alpes  31^st^ Oct 2003—31^st^ Oct 2004  AD |  | 323 |  | 5,184,135 | —  3.5 (3.1-3.9) | —  8.5 (7.9-9.1) | — | 6.1 (5.8-6.4) | 2.43 |
|  |  | Pays de Loire  31^st^ Oct 2003—31^st^ Oct 2004  AD |  | 174 |  | 2,837,379 | —  3.3 (2.8-3.7) | —  8.7 (7.9-9.5) | — | 6.1 (5.6-6.6) | 2.64 |
|  |  | Poitou Charente  31^st^ Oct 2003—31^st^ Oct 2004  AD |  | 91 |  | 1,319,698 | —  3.2 (2.5-3.9) | —  10.0 (8.7-11.1) | — | 6.8 (6.1-7.5) | 3.13 |
|  |  | Ile de France  31^st^ Oct 2003—31^st^ Oct 2004  AD |  | 816 |  | 10,935,798 | —  4.0 (3.7-4.3) | —  9.7 (9.3-10.1) | — | 7.0 (6.7-7.2) | 2.43 |
|  |  | PACA  31^st^ Oct 2003—31^st^ Oct 2004  AD |  | 308 |  | 4,176,207 | —  4.2 (3.7-4.7) | —  10.1 (9.4-10.7) | — | 7.3 (6.9-7.7) | 2.40 |
|  |  | Languedoc Roussillon  31^st^ Oct 2003—31^st^ Oct 2004  AD |  | 134 |  | 1,981,166 | —  3.7 (3.1-4.4) | —  9.1 (8.2-10.0) | — | 6.6 (6.0-7.2) | 2.46 |
|  |  | Corse  31^st^ Oct 2003—31^st^ Oct 2004  AD |  | 15 |  | 210,886 | —  5.6 (3.3-7.9) | —  7.8 (5.2-10.4) | — | 6.8 (5.0-8.5) | 1.39 |
| **Germany** | | | | | | | | | | | |
| [125] | 5/7 | South Lower Saxony  1985  HC/LTC/PA | PD/PO/PP  CA/CR | 16 | 1-year incidence | 265,746 | —  [—] | —  [—] | 6 | — |  |
| [126] | 6/7 | Urban district of Erfurt, Thuringia  1998—2006  HC/LTC/NP/PA/R | PD/PP  CR | 81 | Mean annual incidence | 201,267 | 3.9  [—] | 11.8  [—] | 8.0 (6.39-10.01) | 7.7 (6.08-9.49) | 3.03 |
| **Yugoslavia** | | | | | | | | | | | |
| [130] | 5/7 | Gorksi Kotar, Croatia  1986  AD/HC/NHS/PA | B  CA/CSF/IT/PA | 1 | 1-year incidence | 26,480 | —  [—] | —  [—] | 3.78 | — | — |
| **Croatia** | | | | | | | | | | | |
| [132] | 2/8 | Varazdin County  1960—1997  NR | PNS  NR | 56 | Mean annual incidence | — | —  [—] | —  [—] | 0.795 | — | — |
| **Bosnia & Herzegovina** | | | | | | | | | | | |
| [136] | 4/7 | Western Herzegovina  1994—2003  HC | McD  CR/CSF | — | Mean annual incidence | 300,746 | —  [—] | —  [—] | 1.6 (0-3.3) | — | — |
| [135] | 4/7 | Western Herzegovina Canton & Herzegovina-Neretva Canton  2002—2006  HC | McD  CR/CSF/EP/IT | — | Mean annual incidence | 309,712 | —  [—] | —  [—] | 1.1 (0.6-1.6) | — | — |
| **Romania** | | | | | | | | | | | |
| [11] | 3/7 | Mures County  1986  HC | S  NR | 2 | 1-year incidence | 615,032 | —  [—] | —  [—] | 0.32 | — | — |
| **Greece** | | | | | | | | | | | |
| [139] | 6/7 | Province of Evros  1994—1999  HC/NP/PA/R | PD  CA/CR/CSF/IT/MR | 20 | Mean annual incidence | 143,752 | —  [—] | —  [—] | 2.36 (1.44-3.65) | — | — |
| [138] | 6/7 | Western Greece  2006  HC/PA | McD/PD  CR | 55 | 1-year incidence | 627,058 | 6.64  7.14 (4.41-10.93) | 10.93  12.07 (8.34-16.89) | 8.77 | 9.48 (7.13-12.35) | 1.69 |
| *incidence/100,000 over time period specified (95% CI)  Legend  AD, administrative database; AM, Allison & Millar (definite and/or probable); B, Bauer (1980); CA, Clinical assessment; CR, medical chart review; CSF, cerebrospinal fluid results; DN, documented neurological diagnosis (no criteria specified); HC, hospital or clinic (including allied health) records; ICD, International Classification of Diseases coding; IT, imaging test; LTC, long term care or nursing home facility records; M, media campaign; McA, McAlpine; McD, McDonald 2001; MR, MRI or evoked potentials testing unit records; MS, mailed survey; NHS, national health system records; NP, records of neurologists or other physicians; NR, not reported; PA, patient and other non-governmental associations; PD, Poser Definite; PI, personal interview; PM, post-mortem or other pathological specimen; PNS, Poser not specified; PO, Poser Possible; PP, Poser Probable; R, registry; RO, Rose; S, Schumacher | | | | | | | | | | | |
