# Supplementary material for: Incidence and prevalence of multiple sclerosis in Europe: a systematic review
Source: BMC Neurol. 2013 Sep 26;13:128. doi: 10.1186/1471-2377-13-128 (PMC3856596; doi:10.1186/1471-2377-13-128)
Supplement: Additional file 4: Table S2 — Prevalence of multiple sclerosis, Europe, January 1 1985-January 31, 2011. [file 1471-2377-13-128-S4.doc]

## Additional file 4: Table S2 Prevalence of Multiple Sclerosis, Europe, January 1 1985-January 31, 2011

| **Ref#** | **Quality Score** | **Study Region,**  **Year(s) of prevalence estimate,**  **Sources** | **Diagnostic Criteria, How Established** | **Number cases** | **Mean age (range)**  **years** | **Population or Denominator** | **Crude Prevalence***  **[Standardized Prevalence]***  **Men** | **Crude Prevalence***  **[Standardized Prevalence]***  **Women** | **Crude Overall Prevalence*** | **Age-Standardized Overall Prevalence*** | **Female: Male Ratio** |
| --- | --- | --- | --- | --- | --- | --- | --- | --- | --- | --- | --- |
| **Italy** | | | | | | | | | | | |
| [50] | 5/7 | Town of Sassari, Sardinia  1985  AD/HC/NP/PA | CA | 86 | 35.9 | 124,588 | 38 (24.1-57)  [—] | 98.4 (75.6-126)  [—] | 69 (55.2-85.6) | — | 2.59 |
| [43] | 4/7 | Valle d'Aosta region  1985  HC/NP/PA/R | PD/PP  CA/ | 36 |  | 114,325 | —  [—] | —  [—] | 39 | — | — |
| [13] | 4/7 | City of Catania, Sicily  1989  HC/NP | RO  CA/ | 60 | 40.9 | 380,328 | 15.24  [—] | 16.27  [—] | 15.77 | — | 1.07 |
| [41] | 5/7 | Provinces of Reggio Emilia and Modena, Emilia-Romagna Region  1990,  HC/LTC/NP/PA | McA  CA/ | 404 | 44.4 | 1,024,223 | 23.63 (17.61-30.96)  [—] | 57.72 (47.97-69.38)  [—] | 40.22 (34.46-46.89) | — | 2.44 |
| [51] | 6/7 | Northwestern Sardinia  pre 1985-1993  AD/HC/NP | PD/PP  CA | 276 | 38.3 | 268,926 | 59.2 (46.7-74)  [—] | 144.2 (125.2-166)  [—] | 102.6 (91.5-115.0) | — | 2.44 |
| [40] | 5/7 | Province of Ferrara  Emilia-Romagna region  1993  HC/MR/NP/PA/S | PD/PP  CA/ | 249 | 45.8 | 358,808 | 46.0 (36.4-57.5)  [42] | 90.8 (77.9-105.8)  [85.8] | 69.4 (61.2-78.7) | 64.5 | 1.97 |
| [37] | 5/7 | City of Monreale, Sicily  1991  AD/HC/MR/NP | PD  CA/ | 19 | 45.3 | 26,256 | 62.1  [—] | 82.2  [—] | 72.4 (43.6-113.1) | — | 1.32 |
| [45] | 4/7 | Area of Barbagia , Nuoro, Sardinia  1993  HC/NHS/NP/PA | PD/PP  CA/ | 394 | 41 | 273,768 | 91.6 (76.5-109.65)  [—] | 195.11 (173.65-220.5)  [—] | 143.9 (130.4-158.9) | 149.16 | 2.13 |
| [46] | 5/7 | Area of Barbagia, Nuoro, Sardinia  1994  HC/LTC/NHS/NP/PA | PD/PP  CA/ | 415 | 40.6 | 273,146 | 94.9 (79.5-113.2)  [—] | 207.6 (184.7-234.4)  [—] | 151.9 (137.6-167.7) | 156.4 | 2.19 |
| [36] | 5/7 | City of Bagheria, Sicily  1994  AD/HC/MC/MR/NP | PD  CA/ | 25 | 39.7 | 50,598 | 39.9  [—] | 58.8  [—] | 49.4 (16.2-72.9) | — | 1.47 |
| [53] | 6/7 | District of L'Aquila, Abruzzo region  1996  AD/HC/NP/PA | PD/PP  CA/ | 158 | 38.4 | 297,838 | 36.7 (28.0-48.0)  [—] | 68.4 (56.5-82.8)  [—] | 53 (45.4-62.0) | 55.9 | 1.86 |
| [32] | 5/7 | City of Enna, Sicily  1995  HC/MC/NP/PA | PD  NR | 34 | 37.6 | 28,273 | 110.5  [—] | 129.2  [—] | 120.2 (83.8-167.2) | — | 1.17 |
| [33] | 6/7 | City of Catania, Sicily  1995  HC/NP/PA | PD/PD  CA/ | 195 | 29.6 | 333,075 | 54.8 (44.1-67.9)  [—] | 62.0 (51.1-75.1)  [—] | 58.5 (50.7-67.5) | 55.4 | 1.13 |
| [49] | 5/7 | Province of Sassari, Sardinia  1997  AD/HC/NP/PA | PD/PP  CA/ | 686 | 42.4 | — | 82.5 (71.7-94.9)  [82] | 205.1 (187.8-224.0)  [196] | 144.4 (134.0-155.6) | 140.9 | 2.49 |
| [47] | 5/7 | Province of Nuoro, Sardinia  1998  AD/HC/NP/PA/R | PNS  CA/ | 428 | 39.9 | 272,992 | 93 (81-114)  [—] | 216 (193-243)  [—] | 157 (143-173) | — | 2.32 |
| [42] | 5/7 | Province of Padova  Lombardy region  1999  AD/HC/NP/PA | PD/PP | 667 | 42.3 | 820,318 | 49.7 (41.3-58.1)  [—] | 111.1 (99.0-123.1)  [—] | 80.5 (70.3-90.7) | 81.4 | 2.24 |
| [35] | 5/7 | City of Monreale, Sicily  2000  HC/NP | PD/PP | 21 | 45.3 | 29,493 | 48.5  [—] | 93.0  [—] | 71.2 | — | 1.92 |
| [44] | 5/7 | Province of Genoa, Liguria region  1997  AD/CSF/HC/NP/PA | PD  CA/ | 857 | 48 | 913,218 | 67 (60-76)  [—] | 118 (108-128) | 94 (88-100) | 85 | 1.76 |
| [34] | 6/7 | City of Catania, Sicily  1999  AD/HC/LTC/NP | PD/PD  CA/ | 288 | 31.4 | 313,110 | 69.6 (57.1-84.1)  [80.4] | 89.7 (76.1-105)  [102.4] | 92 (81.8-103.2) | 97.2 | 1.29 |
| [20] | 5/7 | Town of Linguaglossa  Province of Catania, Sicily  2001  AD/HC/LTC/NP/PA | PD/PP  CA/ | 11 | 37.1 | 5,422 | 154.3 (49.1-371)  [—] | 247.4 (108-489)  [—] | 203 (107-352) | 197.8 | 1.60 |
| [38] | 5/7 | Province of Pavia  Lombardy region  2000  HC/NP/PA/R | PD  CA/ | 464 | 41.3 | 493,753 | 68 (59-80)  [—] | 117 (105-132)  [—] | 94 (86-103) | — | 1.72 |
| [31] | 5/7 | City of Caltanissetta, Sicily  2002  HC/NP/PA | PD  NR | 101 | 43.2 | 60,919 | 107.6  [—] | 218.0  [—] | 165.8 (158.5-173.1) | — | 2.03 |
| [39] | 6/7 | Province of Ferrara  Emilia-Romagna region  2004  AD/HC/MR/NP | PD/PP  CA/ | 423 | 49 | 349,777 | 73.59 (61.81-88.01)  [65.15] | 164.26 (144.55-183.97)  [161.18] | 120.93 (110.05-134.23) | 114.28 | 2.23 |
| [54] | 3/7 | Province of Salerno  Campania region  2005  R | PNS | 186 | 44.24 | 259,681 | 43.89 (33.05-57.06)  [—] | 97.49 (80.33-116.6)  [—] | 71.62 (62.03-82.30) | — | 2.22 |
| [52] | 5/7 | Province of Frosinone, Lazio region  2007  AD/HC/NP | PD  CA | 467 | 42 | 491,548 | 53.3 (44.4-63.3)  [—] | 134 (121-150.1)  [—] | 95 (86.6-104.0) | 94.4 | 2.51 |
| **Republic of San Marino** | | | | | | | | | | | |
| [55] | 5/7 | Republic of San Marino  2005  AD/HC/MR/NP/S | PD/PP | 50 | 45.7 | 29,999 | 95.2 (52-160)  [—] | 235.3 (165-327.4)  [—] | 166.7 (123.7-220) | 159.1 | 2.47 |
| **Malta** | | | | | | | | | | | |
| [15] | 4/7 | Malta (Maltese-born residents)  1999  AD/HC/LTC/NP/R | PD/PP | 63 | 43.5 | 378,518 | 13.3  [–] | 19.9  [–] | 16.7 | — | 1.5 |
|  |  | Malta  (Foreign-born Maltese residents  1999  AD/HC/LTC/NP/R |  | 12 | 45.3 | 7,213 | –  [–] | –  [–] | 166 | — | — |
| **England** | | | | | | | | | | | |
| [68] | 5/7 | Borough of Sutton, London  1985  HC/LTC/NP/PA | AM  CR | 195 | 49 | 169,600 | 74 (55-93)  [—] | 152 (126-178)  [—] | 115 (99-131) | 129 (111-147) | 2.05 |
| [57] | 2/7 | Bassetlaw, Nottinghamshire  1988  HC/MS/NP/PA | None used  NR | 98 | — | 100,000 | —  [—] | —  [—] | 98 | — | — |
| [64] | 5/7 | Southampton  South West Hampshire  1987  HC,NP/PA/R | AM/PD/PO/PP  CR/PI | 411 | 48.6 | 417,000 | 64 (54-76)  [—] | 132 (118-148)  [—] | 98.6 (89.3-108.9) | 115 (104-127) | 2.06 |
| [61] | 5/7 | Rural Suffolk  1988  HC/NP/MR/PA | AM  CR | 48 | 49 | 31,379 | —  [—] | —  [—] | 153 (109-196) | — | 2.40 |
| [63] | 5/7 | Cambridge Health District  1990  HC/LTC/NP/PA | AM/PD/PO/PP  CA/CR | 322 | 49.2 | 288,410 | 75 (60-89)  [—] | 184 (162-206)  [—] | 112 | 121 (108-133) | 2.45 |
| [69] | 5/7 | Brighton and Mid-downs  1991  HC/LTC/MR/NP/PA | PD/PP  CR | 665 | 48.6 | 596,594 | 66.2 (57-76)  [—] | 154 (140-168)  [—] | 111 (103-120) | — | 2.33 |
| [65] | 5/7 | North Cambridgeshire  1993  AD/HC/LTC/NP/PA | AM/PD/PO/PP  CR | 449 | — | 379,000 | —  [—] | —  [—] | 118 (108-130) | 139 (127-151) | 2.20 |
| [67] | 6/7 | Rochdale Metropolitan Borough  1989  AD/HC/NP/PA | AM/PD/PO/PP  CA/CR/PI | 232 | 48.9 | 207,600 | 82  [—] | 162  [—] | 122 (107-138) | 143 (126-161) | 1.98 |
| [66] | 5/7 | South Cambridgeshire  1993  HC/LTC/NP/PA/R | AM/PD/PO/PP  CR/PI | 441 | 49 | 290,700 | —  [—] | —  [—] | 152 (138-167) | 179 (164-195) | 2.60 |
| [58] | 5/7 | Leeds Health Authority  1996  HC/NP/PA | PD/PO/PP  CR | 712 | 51 | 732,061 | 52  [—] | 141  [—] | 97.3 | — | 2.71 |
| [62] | 4/7 | London  (3 general medical practices)  1996  AD/HC/NP/PA | NR  CR |  | — | 27,658 | —  [—] | —  [—] | 200 (200-300) | — | — |
| [59] | 5/7 | Leeds Health Authority  1999  NP/PA | PD/PO/PP  CR | 792 | — | 728,840 | —  [—] | —  [—] | 108.7 (101.2-116.5) | — | — |
| [60] | 6/7 | Devon  2001  HC/MR/NP/PA | PD/PP  CR | 402 | 52 | 341,796 | —  [—] | —  [—] | 117.6 (106.1-129.1) | — | 2.65 |
|  |  |  | McD | 323 |  |  |  |  | 117 (105.3-128.2) | — |  |
| **Scotland** | | | | | | | | | | | |
| [21] | 5/7 | Southeast Scotland  1995  HC/LTC/MR/NP | PD/PP  CA/CR | 1613 | 49.2 | 864300 | 112 (102-122)  [—] | 257 (242-272)  [—] | 187 (178-196) | — | 2..29 |
|  |  | Lothian  1995  HC/LTC/MR/NP |  | 1401 | — | 758,600 | —  [—] | —  [—] | 185 (175-194) | 203 (192-214) | — |
|  |  | Border  1995  HC/LTC/MR/NP |  | 212 | — | 105,700 | —  [—] | —  [—] | 201 (174-228) | 219 (191-251) | — |
| [73] | 4/7 | Fife  1996  AD/HC/MS/NP | NR  CR/DN | 208 | 49.7 | 354,273 | 85 (71-99)  [—] | 199 (178-220)  [—] | 143 (131-155) | 178 (164-192) | 2.34 |
| [72] | 6/7 | Tayside  1996  HC/MR/NP | AM/PD/PP  CR | 727 | 49.5 | 395,600 | 100 (86-115)  [85 (73-97)] | 262 (241-285)  [236 (216-256)] | 184 (171-198) | — | 2.62 |
| [74] | 5/7 | Glasgow  1989—1998  HC/NP | DN  CR | 245 | 49.8 | 169,000 | 72 (53-90)  [—] | 213 (202-223)  [—] | 145 (127-163) | — | 2.96 |
| **Wales** | | | | | | | | | | | |
| [77] | 5/7 | County of South Glamorgan  1985  HC/NP/PA | PD/PP  CA/CR/PI | 381 | 48.7 | 376,718 | —  [—] | —  [—] | 101 | — | 1.89 |
| [75] | 4/7 | South East Wales  1988  AD/HC/NP/PA | PD/PP  CR | 379 | — | 376,718 | —  [—] | —  [—] | 100 | — | 2.00 |
| [76] | 5/7 | South East Wales  2005  AD/HC/NP | McD/PD/PP  CA/CR | 620 | 50.8 | 424,633 | 89.1 (77.0-103.1)  [—] | 198.2 (180.5-217.6)  [—] | 146 (135.0-158.0) | 144.6 (133.6-156.5) | 2.82 |
| **Northern Ireland** | | | | | | | | | | | |
| [79] | 4/7 | Coleraine, Ballymoney and Moyle area  NR  AD/HC/NP/PA | AM/PD/PP  CA/CR | 119 | — | 86,500 | —  [—] | —  [—] | 138 | — | — |
| [80] | 5/7 | Ballymoney, Coleraine, Ballymena and Moyle districts  1996  AD/HC/LTC/MS/NP/PA | AM/PD/PO/PP  CA/CR | 254 | 49.3 | 151,000 | 104.1  [—] | 229.9  [—] | 168.2 (147.5-188.9) | — | 2.21 |
| [78] | 6/7 | Ballymoney, Coleraine, Ballymena and Moyle districts  2004  HC/LTC/MS/NP/PA | McD/PD/PP  CA/CR | 370 | — | 160,0446 | 157.0 (130.5-187.4) | 300.8 (264.5-340.8) | 230.6 (207.7-255.4) | 200.5 (193.2-208.0) | 1.24 |
| **Republic of Ireland** | | | | | | | | | | | |
| [81] | 6/7 | County Wexford  2001  AD/HC/LTC/NP/PA | PD/PP  CA/CR | 126 | 47.2 | 104,372 | 88  [—] | 154  [—] | 120.7 (100.6-143.8) | 121.2 | 1.75 |
|  |  | County Donegal  2001  AD/HC/LTC/NP/PA |  | 240 | — | 129,994 | 85  [—] | 282  [—] | 184 (162.0-209.5) | 194.6 | 3.32 |
| **Channel Islands** | | | | | | | | | | | |
| [82] | 5/7 | Guernsey,  1991,  HC/NP/PA | AM/PD/PO/PP  CR | 53 | — | 61,164 | 47.6  [37.3 (17.9-56.7)] | 110.2  [97.5 (73.9-143.5)] | 86.7 (63.3-110.0) | 95.6 (66.9-121.3) | 3.01 |
|  |  | Jersey,  1991,  HC/NP/PA |  | 95 |  | 84,082 | 53.8  [45.5 (26.3-64.7)] | 162  [139.5 (112.6-181.2)] | 113 (90.3-135.7) | 120.2 (96.0-144.3) | 2.32 |
| **Denmark** | | | | | | | | | | | |
| [99] | 8/8 | Denmark,  1990,  R | AM,  CA/MC/MR/PM | — | — | — | —  [—] | —  [—] | 112 | — | — |
| [98] | 6/8 | Denmark,  2001,  AD | ICD,  AD | 9961 | — | 5,472,032 | —  [—] | —  [—] | 182 | — | — |
| [96] | 6/7 | Denmark  2005  R | AM (1994)/PD/PP  CR | 9377 | — | 5,410,000 | 115.6 (111.5-119.7)  [107.0 (100.2-113.7)] | 229.8 (224.1-235.4)  [201.6 (192.4-210.8)] | 173.3 (169.9-176.6) | 154.5 (148.8-160.2) | 1.88 |
| [97] | 6/8 | Denmark  2006  AD | ICD  AD | — | — | 5,506,574 | —  [—] | —  [—] | 189 | — | — |
| **Sweden** | | | | | | | | | | | |
| [94] | 5/7 | Gothenburg region  1988  CSF/HC/NP/R | PD/PO/PP  AD/ | 415 | — | 431,000 | —  [—] | —  [—] | 96 (86.8-105.2) | 84 | — |
| [18] | 4/7 | Overkalix  1990  NP/PA | S  CA/CR | 12 | 53 | 4,744 | 285  [—] | 218 | 253 | — | 0.76 |
| [92] | 5/7 | Vsterbotten  1990  AD/CSF/HC/LTC/MR/NP/PI/R | PD/PO/PP  CA/ | 283 | 47 | 250,134 | 86 (71-104)  [—] | 163 (142-187)  [—] | 125 (112-140) | 126 | 1.90 |
| [93] | 5/7 | Vsterbotten  1997  AD/CSF/HC/LTC/NP | PD/PP  AD/ | 396 | 49 | 259,163 | 105 (89-125)  [—] | 202 (179-228)  [—] | 154 (139-170) | — | 1.92 |
| [95] | 5/7 | County of Varmland  2002  HC/NP | PD/PP  CA/ | 465 | 50.49 | 273,419 | 103.17 (86.1-120.2)  [101.4] | 235.97 (210.3-261.5)  [234.6] | 170.07 (154.5-185.5) | 168.3 | 2.31 |
| **Norway** | | | | | | | | | | | |
| [90] | 4/7 | Counties of More and Romsdal  1985  AD/HC/PA | McA  CR | 179 | — | 237,278 | —  [—] | —  [—] | 75.4 | — | — |
| [86] | 6/7 | Troms and Finnmark  1993  AD/HC | PD/PP/RO  CA/CSF/IT | 184 | 44.9 | 224,724 | 57.8  [—] | 88.7  [—] | 73 (62.3-85.1) | — | 1.53 |
| [84] | 5/7 | Oslo  1995  AD/HC/LTC/NP/PA//R | PD  CA/CSF/IT | 582 | 50.1 | 483,401 | —  [—] | —  [—] | 120.4 (110.9-130.7) | — | 2.10 |
| [85] | 5/7 | Nord-Trondelag County  2000  AD/HC/MC/NP | PD/PP  CA//PI | 208 | — | 127,108 | 122.6 (2.4-7.4)  [—] | 204.8 (171.1-243.2)  [—] | 163.6 (142.2-187.5) | — | 1.67 |
| [88] | 6/7 | Hordaland County  2003  HC/R | PD/PP | 632 | — | 441,660 | 109.8 (96.4-124.3)  [—] | 191.3 (173.5-210.3)  [—] | 150.8 (139.6-162.7) | — | 1.74 |
| [89] | 4/7 | Norway, Ethnic Sami  NR  NP/R | McD/PNS  NR | 12 | — | 40,000 | —  [—] | —  [—] | 30 | — | — |
| [17] | 5/7 | Oslo  2005  NP/R | PD  NR | 786 | — | 529,846 | —  [—] | —  [—] | 148 (138-158) | — | — |
|  |  | Oslo  (Middle Eastern ethnic origin)  2005  NP/R |  | 14 | — | 16,563 | —  [—] | —  [—] | 85 (5.-143) | — | 1.80 |
|  |  | Oslo  (Norwegian/Western ethnic origin)  2005  NP/R |  | 759 | — | 446,335 | —  [—] | —  [—] | 170 (159-182) | — | 2.20 |
|  |  | Oslo  (Asian ethnic origin)  2005  NP/R |  | 9 | — | 42,437 | —  [—] | —  [—] | 21 (11-41) | — | 1.00 |
|  |  | Oslo  (African ethnic origin)  2005  NP/R |  | 4 | — | 20,554 | —  [—] | —  [—] | 20 (7-53) | — | 1.00 |
| **Finland** | | | | | | | | | | | |
| [19] | 5/7 | Vaasa  1993  AD/HC | PD  AD | 156 | — | 179,079 | —  [—] | —  [—] | 107 (90-124) | — | — |
|  |  | Seinajoki-south  1993  AD/HC |  | 219 | — | — | —  [—] | —  [—] | 219(190-247) | — | — |
|  |  | Seinajoki-north  1993  AD/HC |  | 93 | — | — | —  [—] | —  [—] | 136 (108-164) | — | — |
|  |  | Uusimaa  1993  AD/HC |  | 1,052 | — | 1,277,932 | —  [—] | —  [—] | 93 (88-99) | — | — |
| [100] | 5/7 | Central Finland  2000  AD/HC | PD  AD/ | 277 | — | 263,886 | 61 (47-74)  [—] | 148 (127-168)  [—] | 105 (93-117) | — | 2.43 |
|  |  | Central Finland  1993  AD/HC |  | 153 | — | 259,898 | 35 (25-45) | 82 (67-98)  [—] | 59 (50-68) | 58 (50-65) | 2.34 |
| **Iceland** | | | | | | | | | | | |
| [102] | 5/7 | Iceland  1985  AD/HC/NP | PD/PP  CA/ | 185 | — | 240,741 | —  [—] | —  [—] | — | 78.5 (67.9-90.7) | 1.63 |
| [101] | 4/7 | Iceland  1989  HC/NP/PA | PNS  CA | 252 | — | 264,000 | —  [—] | —  [—] | 100 | — | — |
| [103] | 4/7 | Iceland  1999  NP/PA | PNS  CA | 319 | — | 278,717 | —  [—] | —  [—] | 119.12 | — | 2.20 |
|  |  | Iceland  1989  NP/PA |  |  |  | 253,500 |  |  | 113.21 |  | — |
| **Spain** | | | | | | | | | | | |
| [12] | 5/7 | Lanzarote, Canary Islands  1987  HC/NP | PD/PO/PP  CA | 9 | — | 60,000 | [—] | —  [—] | 15 (8.4-24.7) | — | — |
| [14] | 4/7 | Alcoy health region, Valencia  1988  HC/M/NP | RD  CA | 23 | — | 133,915 | —  [—] | —  [—] | 17.17 | — | 2.80 |
| [110] | 5/7 | Sanitary District of Velez, Malaga  1991  HC/M/MR/NHS/NP | PD/PP  CA | 19 | 38.7 | 36,014 | 33  [—] | 74  [—] | 53 (32-82) | 53 | 2.24 |
| [108] | 4/7 | Osona, Catalonia  1991  HC/NHS/NP/LTC/PA | PD/PP  CA/IT | 42 | 43 | 71,985 | 40  [—] | 75.5  [—] | 58 | — | 1.89 |
| [115] | 5/7 | Gijon City, Asturias  1994  HC/LTC/M/MR/NHS/NP/PA | PD/PP  CA | 22 | 38 | 33,775 | 59.9  [—] | 70.2  [—] | 65 (37.9-92.3) | — | 1.17 |
| [111] | 5/7 | Teruel  1996  HC/NP/PA/R | PD/PP  CA | 46 | 40.6 | 143,680 | 23.5 (12.3-34.7)  [—] | 40.6 (25.8-55.4)  [—] | 32 (22.8-41.3) | — | 1.73 |
| [113] | 5/7 | Sanitary District of Calatayud  1995  HC/M/NP/PA | PD  CA | 34 | 43.1 | 58,591 | 37.5 (15.3-59.7)  [—] | 78.5 (46.5-110.6)  [—] | 58 (39-78) | — | 2.09 |
| [107] | 6/7 | Mostoles  1998  HC/ND/NP/PA | PD/PP  CA/CR | 85 | 38.8 | 195,979 | 32.9 (22.6-46.5)  [—] | 53.7 (40.3-70.2)  [—] | 43.4 (34.7-53.7) | — | 1.63 |
| [114] | 5/7 | Valladolid  1997  HC/M/NP/PA | PD/PP  CA | 54 | 36.1 | 92,632 | 40.7  [—] | 74.4  [—] | 58.3 (43.7-75.7) | 53 | 1.83 |
| [118] | 5/7 | Menorca  1996  HC/NP/PA | PD/PP  CA | 46 | 42.1 | 67,009 | 42.1 (23.0-70.7)  [39.9] | 94.7 (64.8-133.7)  [94.1] | 68.6 (50.3-91.6) | 67.3 | 2.25 |
| [117] | 3/7 | La Palma, Canary Islands  1998  HC/NP/PA | PD/PP  CA/CR | 34 | 39.15 | 81,507 | 22  [—] | 60  [—] | 42 | — | 2.73 |
| [112] | 6/7 | Bajo Aragon, Teruel  2003  R | PD/PP  CA | 44 | 41.3 | 58,666 | 50  [—] | 99.7  [—] | 75 (52-97) | 77 (53-98) | 1.99 |
| [116] | 5/7 | Las Palmas City, Gran Canaria Canary Islands  2002  AD/HC/NP/M/MR/NHS/PA | McD/PD/PPCA/CR | 64 | 40.1 | 82,623 | 39.6 (22.6-64.3)  [—] | 113.8 (83.9-150.9)  [—] | 77.5 (59.7-98.9) | 61.6 (47.1-78.9) | 2.87 |
| [106] | 5/7 | Galicia  Santiago de Compostela  2003  HC/M/NP/PA | PD/PP  CA | 10 | 40.5 | 90,188 | 64.1  [—] | 91.6  [—] | 78.7 (60.4-97) | 72 | 1.43 |
| [109] | 3/7 | Malaga (Roma ethnic group)  2002  HC | PD  MCR | 12 | 40 | 22,645 | —  [—] | —  [—] | 52.9 (24-82) | — | — |
| **Portugal** | | | | | | | | | | | |
| [105] | 6/7 | Santarém  1998  HC/LTC/M/NP/PA | PD,  CA | 29 | 42 | 62,621 | 23.3 (6.04-40.5)  [—] | 67.6 (39.3-95.8)  [—] | 46.3 (29.5-63.2) | — | 2.90 |
| **France** | | | | | | | | | | | |
| [120] | 5/7 | Lorraine  2004  AD/HC/NP/PA | PD/PP | 2,718 | — | 2,310,376 | —  [68 (65-72)] | —  [169 (166-172)] | — | 120 (119-121) | 2.60 |
| [123] | 7/8 | Metropolitan France  2004  AD | DN  CA | 49,417 | — | 52,359,912 | 54.8 (54.4-55.3)  [—] | 130.5 (129.8-131.2)  [—] | 94.7 (94.3-95.1) | — | 2.38 |
|  |  | Lorraine  2004  AD |  | 2,507 | — | 2,008,606 | —  [70.8 (68.1-73.5)] | —  [171.5 (167.5-175.5)] | — | 123.7 (121.2-126.2) | 2.42 |
|  |  | Champagne Ardenne  2004  AD |  | 1,348 | — | 1,107,627 | —  [73.4 (69.6-77.1)] | —  [166.5 (161.1-171.8)] | — | 122.9 (119-126.5) | 2.27 |
|  |  | Picardie  2004  AD |  | 1,853 | — | 1,614,238 | —  [65.4 (62.4-68.3)] | —  [163.5 (159.0-167.9)] | — | 116.8 (114.1-119.5) | 2.50 |
|  |  | Bourgogne  2004  AD |  | 1,568 | — | 1,330,427 | —  [62.9 (59.8-66.1)] | —  [162.6 (157.8-167.4)] | — | 116.2 (113.3-119.1) | 2.59 |
|  |  | Franche Comte  2004  AD |  | 1,115 | — | 973,046 | —  [63.2 (59.5-66.9)] | —  [162.2 (156.5-167.8)] | — | 115.3 (111.8-118.7) | 2.57 |
|  |  | Nord pas de Calais  2004  AD |  | 3,949 | — | 3,627,506 | —  [71.7 (69.6-73.7)] | —  [151.3 (148.4-154.2)] | — | 113.3 (111.5-115.1) | 2.11 |
|  |  | Alsace  2004  AD |  | 1,799 | — | 1,597,543 | —  65.8 (62.8-68.7)] | —  [154.6 (150.3-159.0)] | — | 112 (109.4-114.7) | 2.35 |
|  |  | Centre  2004  AD |  | 2,158 | — | 2,077,615 | —  [57.4 (54.9-59.7)] | —  [143.9 (140.3-147.5)] | — | 103.1 (100.8-105.3) | 2.51 |
|  |  | Auvergne  2004  AD |  | 1,118 | — | 1,065,898 | —  [47.1 (44.1-50.1)] | —  [149.7 (144.7-154.8)] | — | 101.5 (98.4-104.5) | 3.18 |
|  |  | Basse Normandie  2004  AD |  | 1,140 | — | 1,179,272 | —  [54.7 (51.5-57.8)] | —  [136.2 (131.4-140.8)] | — | 98 (95.1-100.9) | 2.49 |
|  |  | Haute Normandie  2004  AD |  | 1,510 | — | 1,619,807 | —  [57.2 (54.4-60.0)] | —  [128.7 (124.8-132.7)] | — | 94.7 (92.3-97.1) | 2.25 |
|  |  | Bretagne  2004  AD |  | 2,233 | — | 2,381,323 | —  [52.3 (50.1-54.5)] | —  [131.4 (128.2-134.6)] | — | 94.7 (92.7-96.7) | 2.51 |
|  |  | Limousin  2004  AD |  | 542 | — | 556,268 | —  [43.1 (39.1-47.2)] | —  [136.2(129.6-142.8)] | — | 93.2 (89.2-97.2) | 3.16 |
|  |  | Aquitaine  2004  AD |  | 2,165 | — | 2,416,622 | —  [50.6 (48.5-52.7)] | —  [120.1 (117.1-123.1)] | — | 87.9 (86.0-89.8) | 2.37 |
|  |  | Midi Pyrenees  2004  AD |  | 1,900 | — | 2,158,845 | —  [47.8 (45.6-50.0)] | —  [122.2 (119.0-125.4)] | — | 87.4 (85.3-89.4) | 2.56 |
|  |  | Rhone Alpes  2004  AD |  | 4,450 | — | 5,184,135 | —  [53.5 (52.0-55.0)] | —  [118.2 (116.0-120.2)] | — | 87.3 (86.0-88.6) | 2.21 |
|  |  | Pays de Loire  2004  AD |  | 2,374 | — | 2,837,379 | —  [45.2 (43.3-47.0)] | —  [123.9 (121.0-126.8)] | — | 86.7 (84.9-88.5) | 2.74 |
|  |  | Poitou Charente  2004  AD |  | 1,151 | — | 1,319,698 | —  [46.8 (44.1-49.5)] | —  [118.1 (114.9-123.0)] | — | 85.5 (83-88.1) | 2.52 |
|  |  | Ile de France  2004  AD |  | 9,139 | — | 10,935,798 | —  [50.4 (49.4-51.4)] | —  [115.7 (114.2-117.2)] | — | 84.3 (83.4-85.2) | 2.30 |
|  |  | PACA  2004  AD |  | 3,561 | — | 4,176,207 | —  [49.5 (47.9-51.8)] | —  [114.6 (112.3-116.8)] | — | 84.1 (82.7-85.5) | 2.32 |
|  |  | Languedoc Roussillon  2004  AD |  | 1,655 | — | 1,981,166 | —  [49.5 (47.2-51.8)] | —  [112.0 (108.8-115.3)] | — | 83.1 (81.1-85.2) | 2.26 |
|  |  | Corse  2004  AD |  | 183 | — | 210,886 | —  [50.6 (43.8-57.5)] | —  [108.5 (98.9-118.1)] | — | 81.1 (75.1-87.1) | 2.14 |
| [122] | 7/8 | Haute-Garonne  2004-2005  AD/HC | PD  NR | 1,722 | — | 1,155,838 | —  [—] | —  [—] | 149 | — | — |
| **Belgium** | | | | | | | | | | | |
| [119] | 5/7 | Flanders  1991  HC/NP/PA | PD/PP | 220 | 48 | 250,393 | 73.8 (58.7-89) | 101.3 (80.4-114.5) | 87.9 (76.2-99.4) | — | 1.37 |
| **Switzerland** | | | | | | | | | | | |
| [124] | 4/7 | Berne  1986  HC/NP/PA | PD/PP | 1,016 | 50.7 | 920,000 | 62  [—] | 137  [—] | 110 | — | 2.21 |
| **Germany** | | | | | | | | | | | |
| [125] | 5/7 | South Lower Saxony  1986  HC/LTC/PA | PD/PP  CA/ | 222 | 44.2 | 265,746 | —  [—] | —  [—] | 83.5 | — | — |
| [126] | 6/7 | Urban district of Erfurt, Thuringia  2006  HC/LTC/NP/PA/R | PD/PP | 256 | — | 201,267 | —  [—] | —  [—] | 128 | — | — |
| **Austria** | | | | | | | | | | | |
| [127] | 1/7 | Austria  1998—1999  NP/PA | PD | 7,982 | — | 8,078,400 | —  [—] | —  [—] | 98.8 | — | — |
| **Hungary** | | | | | | | | | | | |
| [129] | 4/7 | Szeged  1996  HC | PD/PP | 130 | — | 198,682 | —  [—] | —  [—] | 65 | — | 3.00 |
| [128] | 3/7 | Csongrad County  1999  HC/LTC/NP | PD/PP  CA | 248 | — | 400,128 | —  [—] | —  [—] | 62 | — | 2.75 |
| **Yugoslavia** | | | | | | | | | | | |
| [130] | 5/7 | Gorksi Kotar, Croatia  1986  AD/HC/PA | B  CA/CSF/IT/PI | 38 | 39.9 | 26,480 | —  [—] | —  [—] | 143.5 (101.6 -196.6) | — | 1.92 |
| [131] | 5/7 | Belgrade  1996  HC | PD  CA/ | 823 | — | 1,602,226 | —  [28.2 (24.5-32.5)] | —  [54.1 (49.5-59.1)] | 44.9 | 41.5 (38.5-44.7) | 1.92 |
| **Croatia** | | | | | | | | | | | |
| [133] | 5/7 | Varazdin County, Croatia  1997  AD/HC/PA | PNS  NR | 56 | — | 190,203 | —  [—] | —  [—] | 29.44 (22.68-38.23) | — | — |
|  |  | Varazdin County, Croatia  1991  AD/HC/PA |  | 45 | — | 187,853 | 15.37 (9.15-25.79)  [—] | 32.28 (22.74-45.81)  [—] | 24.04 (17.97-32.17) | — | 2.1 |
| **Croatia /Slovenia** | | | | | | | | | | | |
| [134] | 5/7 | Kocevje and Gorski Kotar region (Slovenia and Croatia)  NR  AD/HC/NP/PA | PD  CA/ | 87 | 47.6 | 57,258 | 127.6 | 175.7 | 151.9 (123.2-187.4) | — | 1.38 |
| **Bosnia and Herzegovina** | | | | | | | | | | | |
| [136] | 4/7 | Western Herzegovina  2003  HC | McD  CSF/IT | 81 | 40 | 300,746 | 23 (14-33)  [—] | 30 (20-40)  [—] | 26.9 (19.9-33.9) | 26.09 | 1.3 |
| [135] | 4/7 | Western Herzegovina Canton & Herzegovina-Neretva Canton  2006  HC | McD5  CSF/T | 96 | 41.2 | 309,712 | —  [—] | —  [—] | 30.99 (24.8-37.2) | — | 1.5 |
| **Bulgaria** | | | | | | | | | | | |
| [137] | 4/7 | Svoge  1995  HC/NP | PD/PP  CA/CSF/IT | 9 | 43 | 22,913 | —  [—] | —  [—] | 39.3 | — | — |
|  |  | Trojan  1995  HC/NP |  | 12 | 43.7 | 30,660 | —  [—] | —  [—] | 39.1 | — | — |
| [16] | 5/7 | Sofia (non-Roma & Roma)  1998  AD/HC/NP | PD/PP  CA/CSF//IT | 32 | 41.2 | 74,334 | —  [—] | —  [—] | 43.05 (29.45-60.78) | — | — |
|  |  | Sofia (non-Roma)  1998  AD/HC/NP |  | 31 | — | 69,094 | —  [—] | —  [—] | 44.87 (30.50-63.68) | — | — |
|  |  | Sofia (Roma)  1998  AD/HC/NP |  | 1 | — | 5,240 | —  [—] | —  [—] | 19.08 (0.98-106.30) | — | — |
|  |  | Samokov (non-Roma & Roma)  1998  AD/HC/NP |  | 17 | 45.6 | 44,616 | —  [—] | —  [—] | 38.10 (22.12-61.01) | — | — |
|  |  | Samokov (Non-Roma)  1998  AD/HC/NP |  | 15 | — | 33,747 | —  [—] | —  [—] | 44.45 (24.89-73.31) | — | — |
|  |  | Samokov (Roma)  1998  AD/HC/NP |  | 2 | — | 10,869 | —  [—] | —  [—] | 18.40 (2.23-66.43) | — | — |
| **Romania** | | | | | | | | | | | |
| [11] | 3/7 | Mures County  1986  HC | S  NR | 139 | — | 615,032 | 18.08  [—] | 23.8  [—] | 20.97 | — | 1.32 |
| **Greece** | | | | | | | | | | | |
| [139] | 6/7 | Province of Evros  1999  HC/NP/PA | PD,  CA/CSF/IT | 56 | — | 143,752 | —  [—] | —  [—] | 38.9 | 38.32 | 2.8 |
| [138] | 6/7 | Western Greece  2006  HC/PA | McD/PD  CR | 780 | 38.04 | 652,108 | 98.81  [—] | 141.05  [—] | 119.61 | — | 1.43 |
| **Cyprus** | | | | | | | | | | | |
| [24] | 5/7 | Greek speaking districts (Paphos, Famagusta and “The Mountains”)  1988  HC/NP/PA/PI | PD/PO/PP  CA/CSF/IT | 42 | — | 108,600 | 36.9  [—] | 29.1  [—] | 38.7 (27.9-52.3) | — | 1.06 |
|  |  | Paphos district  1988  HC/NP/PA/PI |  | 22 | — | 49,500 | —  [—] | —  [—] | 44.4 | — | — |
|  |  | Famagusta District  1988  HC/NP/PA/PI |  | 10 | — | 29,100 | —  [—] | —  [—] | 34.4 (16.5-63.6) | — | — |
|  |  | "The Mountains"  1988  HC/NP/PA/PI |  | 10 | — | 30,000 | —  [—] | —  [—] | 33.3 (16.0-61.2) | — | — |
| [23] | 4/7 | Turkish Cypriots  1993  AD/HC/NP/PI | PD/PP  CA/ | 71 | — | 176,000 | 54  [—] | 27  [—] | 40 | — | 0.5 |
|  |  | Greek Cypriots  Nicosia Metropolitan area  1993  AD/HC/NP/PI |  | 24 | — | 46,990 | 42.9  [—] | 59.1  [—] | 51.1 | — | 1.38 |
| **Estonia** | | | | | | | | | | | |
| [140] | 4/7 | South Estonia  1989  HC/LTC/NP/PA | S  CA//PI | 200 | 44 | 392,009 | 36.8  [—] | 63.3  [—] | 51.0 | 49.56 | 1.72 |
|  |  | South Estonia (Estonians)  1989  HC/LTC/NP/PA |  | — | — | — | —  [—] | —  [—] | 55 | — | — |
|  |  | South Estonia (Russians)  1989  HC/LTC/NP/PA |  | — | — | — | —  [—] | —  [—] | 29 | — | — |
| * prevalence/100,000 (95% CI)  Legend  AD, administrative database; AM, Allison & Millar (definite and/or probable); B, Bauer (1980); CA, Clinical assessment; CR, medical chart review; CSF, cerebrospinal fluid results; DN, documented neurological diagnosis (no criteria specified); HC, hospital or clinic (including allied health) records; ICD, International Classification of Diseases coding; IT, imaging test; LTC, long term care or nursing home facility records; M, media campaign; McA, McAlpine; McD, McDonald 2001; MR, MRI or evoked potentials testing unit records; MS, mailed survey; NHS, national health system records; NP, records of neurologists or other physicians; NR, not reported; PA, patient and other non-governmental associations; PD, Poser Definite; PI, personal interview; PM, post-mortem or other pathological specimen; PNS, Poser not specified; PO, Poser Possible; PP, Poser Probable; R, registry; RO, Rose; S, Schumacher | | | | | | | | | | | |
